# Comprehensive analysis of genes associated with migraine in the Indian population: a meta-analysis of genetic association studies with trial sequential analysis

**DOI:** 10.1038/s41598-023-45531-3

**Published:** 2023-11-04

**Authors:** Amrit Sudershan, Agar Chander Pushap, Meenakshi Bhagat, Isha Sharma, Hardeep Kumar, Sanjeev K. Digra, Parvinder Kumar

**Affiliations:** 1 Institute of Human Genetics, University of Jammu, Jammu, Jammu and Kashmir 180006 India; 2 Department of Human Genetics, Sri Pratap College, Cluster University of Srinagar, Kashmir, Jammu and Kashmir India; 3 Department of Education, Dakshina Bharat Hindi Prachar Sabha, Madras, 600017 India; 4 Department of Zoology and Institute of Human Genetics, University of Jammu, Jammu, Jammu and Kashmir 180006 India; 5 Department of Neurology, Super Specialty Hospital, Jammu, Jammu and Kashmir 180006 India; 6 Department of Paediatrics, Sri Maharaja Gulab Singh Hospital, Government Medical College, Jammu, Jammu and Kashmir 180006 India

**Keywords:** Genetics, Genetic association study, Neurology, Headache, Migraine

## Abstract

Migraine is a complex disorder with multigenic inheritance and is characterized by the cardinal symptom of unilateral headache. Many genes are responsible for increasing the susceptibility of disease within different populations. Therefore, our primary aim in this review was to catalog the many genes that have been studied in India and after collecting the necessary information, we calculated a more precise risk relationship between an identified variation and migraine. The gene and its associated risk variant were discovered in the Indian population using a PRISMA-based systematic literature review guideline from online databases such as PubMed & Google Scholar. We constructed pooled odds ratios with 95% confidence intervals using multiple genetic models. Also, we looked for heterogeneity using Cochran's Q Test and the I2 statistic. Publication bias was analyzed using Begg's and Egger's tests. A p-value less than 0.05 was judged to be statistically significant for all tests. After a critical analysis, a total of 24 studies explored about 21 genes with 31 variants out of which only nine genes have been studied more than two times in the Indian population and thus were found eligible for the meta-analysis. It has been found, that the *ACE-*DD variant (allele model: OR: 1.37 [1.11–1.69], I^2^ = 0%/ fixed model), *ESR1-*PvuII (allele model: OR: 1.47 [1.24–1.74], I^2^ = 0%/ fixed model) significantly increases the risk of migraine in Indian population. Also, a protective role of the *LRP1*-rs11172113variant was observed for both migraine and its clinical subtype i.e., MA (allelic model: OR of 0.65 [0.50–0.83] I^2^ = 44% and allele: OR: 0.54 [0.37–0.78], I^2^ = 52%) respectively. Overall, the results of this meta-analysis indicated that the ACE-DD variant and the ESR1-PvuII were associated with an increased risk of migraine in the Indian community, while the LRP1-rs11172113 variant was associated with protection from migraine in this population.

## Introduction

Migraine is a complex and polygenic disorder, featuring different characteristics such as nausea, vomiting, phonophobia, photophobia, and interestingly the cardinal feature i.e., unilateral headache^1^. International Classification of Headache Disorder 3rd edition (ICHD-3) has classified the disorder into two main clinical subtypes i.e., Migraine with Aura (MA) and Migraine Without Aura (MWA) based on the criteria of presence and absence of aura feature (ICHD-3.org/1-migraine/). Various factors, such as cortical spreading depression (CSD), activation and desensitization of the Trigeminal-vascular system, neurogenic-neuroinflammation, etc., which are collectively responsible for the etiology of migraines, have previously been explored^2–6^. Factors that are responsible for increasing the risk of migraine are crucial and are broadly categorized into environmental and genetic factors. Former which is responsible for hindering the susceptibility threshold of pain which is set by the latter factor i.e., genetic risk factors^1^.

Genetic risk factors include genes with changes/variations in the gene sequence with a major type called Single Nucleotide Polymorphism (SNP) or Single Nucleotide Variation (SNV) which are responsible for altering the function of the same. The most recent and updated meta-analysis of the Genome-Wide Association Study (GWAS) data has shown that many genes with modest effects are involved in disease risk^7^. Other than the advanced GWAS, numerous independent studies have been carried out in various populations and have identified various genes that are responsible for disease risk attribution. Using India as an example of a population, which is part of the Asian ethnic group where migraine disorder is very common^8–12^, many genes have been studied (Table 1) but the association between these genes and migraine risk has been found to be inconsistent^13–36^.

**Table 1:** Multiple gene identified in the population of India.

S. No	Gene	Protein	Chr	Function	SNPs	Diagnosis	Case/controls	State	Region	ReL	Comment	Ref
1	ACE	Angiotensin I converting enzyme	17q23.3	It catalyzes the conversion of angiotensin I into a physiologically active peptide angiotensin II. Angiotensin II is a potent vasopressor and aldosterone-stimulating peptide that controls blood pressure and fluid-electrolyte balance	I/D	IHS	100/121	Kashmir	NI	X	We were not able to find a statistically significant association between ACE gene I/D polymorphism with migraine	Wani et al.^24^
	ACE				I/D	IHS	102/150	Jammu	NI	✓	ACE I/D polymorphism analysis revealed that D allele and DD genotype is considerably associated with the risk for migraine in population of North India (Jammu region)	Jasrotia et al.^17^
	ACE				I/D	IHS	150/150	Lucknow, UP	NI	✓	ACE DD genotype showed significant association in migraine patients	Joshi et al.^13^
2	ANKK1	Ankyrin repeat and kinase domain containing 1	11q23.2	A TaqIA polymorphism (rs1800497) is located 9.5 kb downstream from DRD2 gene. It causes an amino acid change (Glu713Lys) in the C-terminal ankyrin repeat domain of ANKK1 (ankyrin repeats and kinase domain containing 1) gene	rs1800497		335/200	Lucknow, UP	NI	✓	The ANKK1 polymorphism, variant genotype and allele showed significant associations with migraine risk	Ghosh et al.^27^
3	APOE	Apolipoprotein E protein coding	19q13.32	APOE ε4 increases the uptake of arginine in microglia as compared to APOE ε3 and thus may regulate production of nitric oxide	E2		50/50	New Delhi	NI	✓	APOE ε2 gene increases the risk of migraine	Gupta et al.^32^
	APOE				HhaI		217/217	Lucknow, UP	NI	✓	After performing Bonferroni correction and setting the standard p-value to be 0.008, E3E4 and E2E3 when taken together conferred risk in the case of migraine versus HC	Joshi et al.^33^
4	CACNA1A	Calcium voltage-gated channel subunit alpha1 A	19p13.13	Voltage-dependent calcium channels mediate the entry of calcium ions into excitable cells, and are also involved in a variety of calcium-dependent processes, including muscle contraction, hormone or neurotransmitter release, and gene expression	rs16023/ E993V A > T		25/25	Kashmir	NI	X	In our study, we could not find any polymorphism of CACNA1A gene in the selected patient	Bashir et al.^31^
5	DAO	D-Amino acid oxidase	12q24.11	Encodes the peroxisomal enzyme D-amino acid oxidase which is a flavoprotein and uses flavin adenine dinucleotide (FAD) as its prosthetic group. Its substrates include a wide variety of D-amino acids, but it is inactive on the naturally occurring L-amino acids	rs10156191	IHS	25/250	New Delhi	NI	✓	rs10156191T is found to be significantly associated with the risk of migraine in North Indian population	Kaur et al.^20^
	DAO				rs10156191	IHS	100/100	Tamil Nadu	SI	✓	rs10156191 seem to be associated factors with the risk of migraine	Thomas et al.^35^
	DAO				rs2052129	IHS	250/250	New Delhi	NI	✓	The increase of variants rs2052129T in patients of New Delhi of North India may be related with reduced DAO gene activity and thus risk of migraine	Kaur et al.^20^
6	DBH	Dopamine beta-hydroxylase	9q34.2	Encoded protein is an oxidoreductase belonging to the copper type II, ascorbate-dependent monooxygenase family and are expressed in neuroscretory vesicles and chromaffin granules of the adrenal medulla, catalyzes the conversion of dopamine to norepinephrine, which functions as both a hormone and as the main neurotransmitter of the sympathetic nervous system	19 bp I/D	IHS	301/202	Lucknow, UP	NI	✓	A significant association was observed at allelic level (P = 0.027) especially in females (P = 0.016) on comparing migraine patients with healthy controls	Ghosh et al.^36^
	DBH				rs1611115	HIS	335/200	Lucknow, UP		X	We were unable to find any significant associations of rs1611115 polymorphism with migraine	Ghosh et al.^27^
	DBH				rs7239728	IHS	335/200	Lucknow, UP		✓	DBH rs7239728 imparted significant risk at genotypic, allelic and carrier analyses	Ghosh et al.^26, 27^
7	DRD2	Dopamine receptor D2	11q23.2	Encoded protein is a D2 subtype of the dopamine receptor which act as a G-protein coupled receptor inhibits adenylyl cyclase activity and it might be involved in central sensitization and regulate AMPARs in chronic migraine	NcoI		301/202	Lucknow, UP	NI	X	Not significant association	Ghosh et al.^27^
	DRD2				rs1799732		335/200	Lucknow, UP		P	Protective effect	Ghosh et al.^26, 27^
	DRD2				rs6275		335/200	Lucknow, UP		P	Protective effect	Ghosh et al.^26, 27^
8	EDNRA	Endothelin receptor type A	4q31.22-q31.23	Encoded protein act as a receptor for endothelin-1, a peptide that plays a role in potent and long-lasting vasoconstriction. This receptor associates with guanine-nucleotide-binding (G) proteins, and this coupling activates a phosphatidylinositol-calcium second messenger system	-231 G > A		217/217	Lucknow, UP	NI	✓	We found an association of EDNRA -231 G > A polymorphism with migraine subjects as well as migraine without aura	Joshi et al.^33^
9	ESR1	Estrogen receptor 1	6q25.1-q25.2	Encoded protein is an estrogen receptor and ligand-activated transcription factor with an N-terminal ligand-independent transactivation domain, a central DNA binding domain, a hinge domain, and a C-terminal ligand-dependent transactivation domain. It regulates cortical excitability and spreading depression which is an important pathophysiological mechanism of migraine	PvuII	ICHD-3	102/115	Jammu	NI	X	A significant association of ESR-XbaI polymorphism was observed with migraine	Kumar et al. 2022
	ESR1				PvuII		217/217	Lucknow, UP	NI	✓	A significant association ESR1 PvuII single nucleotide polymorphism with migraine when compared with HC	Joshi et al.^14^
	ESR1				rs180112/ C325G		217/217	Lucknow, UP	NI	X	No significance of ESR 325 G → C polymorphism was seen in any of the models under study	Joshi et al.^14^
	ESR1				XbaI	ICHD-3	102/115	Jammu	NI	✓	We found significant association between ESR-XbaI polymorphism and migraine susceptibility	Kumar et al. 2022
	ESR1				XbaI		334/200	Lucknow, UP	NI	X	Only the studied variant found to be associated with MA	Ghosh et al.^37^
10	LRP1	Lipoprotein receptor-related protein 1	12q13.3	LRP may interact with neuronal glutamate receptors, thus also providing a link to the glutamate pathway which is a critical neurotransmitter pathway linked to cortical hyperexcitability	rs11172113	IHS	340/200	Lucknow, UP	NI	P	Protective	Ghosh et al.^26^
	LRP1				rs11172113	-	45/58	Tamil Nadu	SI	X	Not significant association	Shoba et al.^28^
	LRP1				rs11172113	ICHD-3	100/100	New Delhi	NI	X	Not significant association	Kaur et al.^21^
11	MTHFR	Methylenetetrahydrofolate reductase	1p36.22	The protein encoded by this gene catalyzes the conversion of 5,10-methylenetetrahydrofolate to 5-methyltetrahydrofolate, a co-substrate for homocysteine remethylation to methionine and a circulating form of folate in plasma which is needed for the conversion of homocysteine to methionine	A1298C		186/152	Vellore, Tamil Nadu	SI	✓	Similarly, it was found that CC 1298 was also associated with more Migraineurs	Thomas et al.^23^
	MTHFR				A1298C	ICHD-3	100/100	New Delhi	NI	✓	CC genotype in A1298C was found to be a risk factor in migraine patients than controls	Kaur et al.^18^
	MTHFR				C677T	IHS	186/152	Vellore, Tamil Nadu	SI	✓	Interestingly, TT of genotype 677 was significantly correlated with Migraine	Thomas et al.^23^
	MTHFR				C677T	IHS	204/210	Tamil Nadu	SI	✓	The case–control study confirms a positive association between MTHFR rs1801133 (C/T) polymorphism and migraine susceptibility in South Indian population	Aiswarya et al.^22^
	MTHFR				C677T	IHS	100/120	Kashmir	NI	X	MTHFR 677TT is not associated with a risk for the development of migraine in our group of patients from our population	Pandith et al.^16^
	MTHFR				C677T	ICHD-3	102/150	Jammu	NI	✓	MTHFR 677 CT genotype and variant T allele only in cases but not in controls which suggests a possible involvement of variant T allele in migraine development	Jasrotia et al.^17^
	MTHFR				C677T	IHS	100/100	New Delhi		X	A non-significant increase in frequencies of CT and TT in C667T SNP in migraine patients with control	Kaur et al.^18^
	MTHFR				C677T	IHS	150/150	Lucknow, UP	NI	X	No significant differences in genotype and allelic frequencies of MTHFR C677T polymorphism were found on comparing migraine patients with either disease controls or healthy controls	Joshi et al.^13^
12	PRDM16	PR/SET domain 16	1p36.32	Encoded protein is a zinc finger transcription factor and contains an N-terminal PR domain. The potential role of *PRDM16* is still inexact in migraine pathophysiology	rs2651899	IHS	150/150	New Delhi	NI	✓	rs2651899 is a potential genetic marker for migraine susceptibility in MO and female subgroup at both genotypic and allelic level in the North Indian population	Kaur et al.^19^
	PRDM16				rs2651899	IHS	340/200	Lucknow, UP	NI	P	Protective	Ghosh et al.^26^
13	PROGINS/ PGR	Progesterone receptor	11q22.1	Encoded protein mediates the physiological effects of progesterone, and interaction with ESR1 showed a synergistic effect and found to be increased the risk of migraine	rs1042838		217/217	Lucknow, UP	NI	P	Significant differences in genotypic and allelic frequency were seen in case of PROGINS polymorphism when migraine patients were compared with HC, showing a protective effect	Joshi et al.^14^
14	SLC6A4/SERT	Solute carrier family 6 member 4 /serotonin transporter	17q11.2	Encoded protein act as a serotonin transporter (5-HTT) which help in the synaptic clearance of 5-HT but due to variant such as 5-HTTLPR (5-HTT Linked Polymorphic Region) leads to the half the number of serotonin transporters thus slower synaptic clearing and accounts for altered serotonin levels and may thus be associated with migraine	102 T > C		217/217	Lucknow, UP	NI	X	We did not find any significant differences in the frequencies of genotypes in case of HT 102 T > C polymorphism when migraine patients were compared with healthy controls	Joshi et al.^15^
	SLC6A4/SERT				5HTTLPR	IHS	304/308	Tamil Nadu	SI	X	The genotyping analysis revealed insignificant relationship with migraine subjects when compared with controls	Kesavan et al.^34^
	SLC6A4/SERT				STin2 VNTR		217/217	Lucknow, UP	NI	X	No significant difference in genotype and allele frequencies was observed between migraine patients and healthy control	Joshi et al.^15^
15	TGFBR2	Transforming growth factor beta receptor 2	3p24.1	encoded protein is a transmembrane protein that has a protein kinase domain, forms a heterodimeric complex with TGF-beta receptor type-1, and binds TGF-beta and it has been found that highly enriched in vascular tissues and metal ion homeostasis suggesting the important role in migraine pathophysiology	rs7640543		340/200	Lucknow, UP	NI	X	Not significant association	Ghosh et al.^26, 27^
16	TNF-A	Tumor necrosis factor-alpha	6p21.23	Encoded protein is an multifunctional proinflammatory cytokine mainly secreted by microglial cells in brain and is considered as an prime candidate for neurogenic neuroinflammation	rs1800629		212/218	Tamil Nadu	SI	✓	The results of this case–control study discovered a significant relationship with Mg in recessive and homozygous genotype	Kesavan et al.^30^
	TNF-A				rs1800629		216/216	Lucknow, UP	NI	✓	A borderline association was observed in TNFA 308GA genotype in migraine patients versus HC	Ghosh et al.^25^
17	LT-α/ TNF-B	Lymphotoxin-alpha /tumor necrosis factors- beta	6p21.33		G252A		216/216	Lucknow, UP	NI	X	We could not find any association of TNFB 252G > A polymorphism in genetic susceptibility to migraine on comparing the migraine patients with HC	Ghosh et al.^25^
18	TRPM8	Transient receptor potential cation channel subfamily M member 8	2q37.1	Encoded protein enable the ligand-gated calcium channel activity and involved in calcium ion transmembrane transport. It is expressed on sensory afferents innervating the meninges, and these neurons are subject to developmental changes that may influence their contribution to migraine	rs10166942		340/200	Lucknow, UP	NI	X	Not significant association	Ghosh et al.^26^
19			8q21	–	rs10504861		100/100	New Delhi	NI	X	Not significant association	Kaur et al.^21^
20	LOC101927066	Uncharacterized LOC101927066	8q22.1	–	rs1835740		340/200	Lucknow, UP	NI	X	We did not observe any significant effect of the variant genotype or allele of the first migraine GWAS associated marker, rs1835740	Ghosh et al.^26^
21	SUGCT	Succinyl-CoA:glutarate-CoA transferase	7p14.1	–	rs4379368		100/100	New Delhi	NI	X	Not significant association	Kaur et al.^21^

*NI* North India, *SI* South India, *UP* Uttar Pradesh, *X* Not significantly associated, ✓ significantly associated, *ReL* relationship.

As a result, in the current review study, we first sought to identify the different genes explored in the Indian population before pooling the risk of similar genes to determine the precise association in the same population. The current study is unique in that it is the first of its kind to include all of the risk genes and variants from the Indian population to determine the precise risk.

## Method

### Literature survey

The presented review aimed to find out the critical genes that increase the risk of migraine and its clinical subtype in the population of India which belongs to the Asian ethnic group. To achieve the aim, we used the approach of a “systematic way of literature survey” which was done from the online database and search engines such as PubMed-NCBI (National Center for Biotechnology Information) (Pubmed.NCBI.nlm.nih.gov), and Google Scholar (Scholar.google.com.tw) respectively. We bypassed exploring other databases and utilized PubMed because of its comprehensive collection of references, which includes MEDLINE (Medical Literature Analysis and Retrieval System Online), life science journals, and electronic books.

Multiple key terms were used in our search strategy, including “Gene polymorphism with migraine in India *OR* Gene variant with migraine in India *AND* migraine genes in India *OR* migraine polymorphism in India”. Only articles published in the English language were evaluated using the linguistic filters as a factor for publication selection. We also tried to exclude study data if unpublished (Research Square/ Researchsquare.com), incomplete, or only partially available. Because partial and missing data are not included in the study, there is no such detrimental effect and we did our utmost to eliminate any undesirable characteristics. The search was completed and studies were included following the PRISMA (Preferred Reporting Items for Systematic Reviews and Meta-Analyses) guidelines (www.prisma-statement.org) (Supplementary Prisma Check List-S1)^38^.

### Inclusion and exclusion features

Concerning the study inclusion criteria, the following inclusion criteria should be met and those include “A case–control or cohort study design must be the prime requirement followed by the “the study must represent the Indian population criteria” for the screening of study”, second the “authors must have investigated/ diagnosed the patients according to the criteria of the International Headache Society (IHS) or ICHD-3″ International Classification of Headache Disorders-3, the authors must have looked at the genetic polymorphisms/variants and must have provided the detailed description about the variant under study with proper reference ID (rsID) or change of nucleotide”, “the genotype frequencies of the investigated variants must be stated unambiguously among migraineurs and controls”, “Hardy–Weinberg Equilibrium (HWE) conditions are required for all experiments”, “studies should provide clear data to calculate the odds ratios (ORs) and the corresponding 95% confidence intervals (CI)”.When necessary information was missing from an article, it was sourced from another publication.

### Data extraction

The demographic characteristics of each study were extracted, including the state in which the research was conducted, the number of patients and healthy individuals, any cohort data, the genotypic frequency from both cases and controls, the first authors, the years of publication, and the technique used to determine the genotype? Also features such as gene coding protein, chromosomal location, the function of a protein, and polymorphism ID/SNP-ID/rsID were extracted from the online data where required. If any statistical/numerical data were found missing, previous research/references were analyzed. All the data/features extraction was first done by our two authors (A.S, A.C.P, M.B, & I.S) and then the quality was assessed by the other two authors (A.S, A.C.P, & H.K).

### Quality assessment

The quality of research articles is an important factor to consider when performing a meta-analysis, which entails pooling independent research studies to determine the precise result. In light of this, the present research assessed the quality of all previously published studies by utilizing the criteria established by the Newcastle–Ottawa scale (NOS) such as (1): selection of cases and controls that include cases and control definition, and their selection(2): comparability (comparability of cases and controls) and (3): ascertainment of exposures (exposure ascertainment, case, and control ascertainment, and non-response rate). Each section with the correct method is assigned one star (1 point) with an exception in Comparability” section which is with two stars (2 points). Therefore, a study will be disqualified if it receives fewer than 5 stars (< 5) (or 5 points), which is considered to be a good study and can only receive a maximum of 9 stars (Ottawa Hospital Research Institute (ohri.ca) (Supplementary File: SF1-NOS). If any differences in decision-making were noticed concerning article inclusion, data extraction, or quality assessment/ NOS, the third investigator (P.K) investigated and concluded the matter.

### Statistical analysis

Genotypic and allelic frequency was first calculated for all studies included in the meta-analysis and then the Chi-square test was used to analyze whether the population is in HWE (p > 0.05 for the population in HWE) or not (p < 0.05). To find out the strength of the association between the variant of interest, and the risk of migraine, the logistic regression utilizing OR (Odds Ratio) model with 95% CI (Confidence Interval) was used. Here odds ratio is defined as the OR > 1 is defined as the odds of exposure among cases being greater than the odds of exposure among controls & OR < 1: the odds of exposure among cases are lower than the odds of exposure among controls. Different genetic models such as allelic (rare allele vs. wild allele), dominant (dominant vs. heterozygote + pure recessive), recessive (recessive vs. dominant + heterozygote), and over-dominant (heterozygote vs. pure dominant + recessive) were used to observe the strength of association (OR) using random: Dersimonian and Laird method or fixed model (Inverse variance method) based on I^2^(I^2^ > 75: Random model). I^2^ is an estimate to define the proportion of inter-study variability attributed to chance rather than heterogeneity.

The publication bias including reporting bias and heterogeneity of the research studies were assessed using Begg's and Egger's tests and χ2 based on Cochran’s Q Test with I-square (I^2^) tests respectively. Also, we performed the sensitivity analysis to observe the influence of individual studies on the pooled ORs and 95% CIs by the criteria of “exclusion of each study”.All tests were two-sided, and a value < 0.05 was considered statistically significant. The current meta-analysis process, from the choice of statistical tests to the analysis of the findings, was conducted following the Cochrane guidelines (Training.cochrane.org/handbook/current). All the statistical analysis was done with Meta-Genyo online Statistical Analysis System software (MetaGenyo: Meta-Analysis of Genetic Association Studies).

### Trial sequential analysis

To reduce the possibility of random error, the current meta-analysis makes use of a method called Trial Sequential Analysis (TSA), which checks to see if the included trials have sufficient numbers of participants. Based on an overall risk of 5% and a relative risk reduction of 20% (with 80% power), the TSA tool (Copenhagen Trial Unit, Denmark) was used to compute the necessary information size for evaluating the validity of a meta-analysis. (TSA—ctu.dk). With specific settings “Set Effect Measure and model” (Effecet Measure: Odds Ratio, Model: Fixed), “Set Zero Event Handling (SZEH)” (Method: Constant, Value: 1.0, Included trials with no events: checked), “Set Confidence Interval (SCI)” (Conventional/ Coverage: 95%). There are, however, two basic possibilities: (1) no further research is needed if the cumulative Z value/curve exceeds the RIS (Required Information Size); (2) additional studies are needed if the Z curve does not surpass the RIS threshold.

### Protein–protein interaction

It is critical to identify the most important wiring connection/most connected node/dot in the gene/protein interaction network used to understand disease progression because any change in the peripheral gene will eventually affect the regulation of the core gene/ most connected node^39^. Therefore, to analyze the most connected gene/node among the studied genes in the respective population, the String v11 (String-db.Org/), a potential protein–protein interaction tool that collects data from several online databases was used. After PPI (Protein–Protein Interaction), the network was built, edited, and analyzed using the Cytoscape tool version 3.9.1, a free and publicly available bioinformatics tool for analyzing and interpreting gene expression profiles, and molecular interaction networks (Cytoscape.org/).

## Result

**Figure 1:**
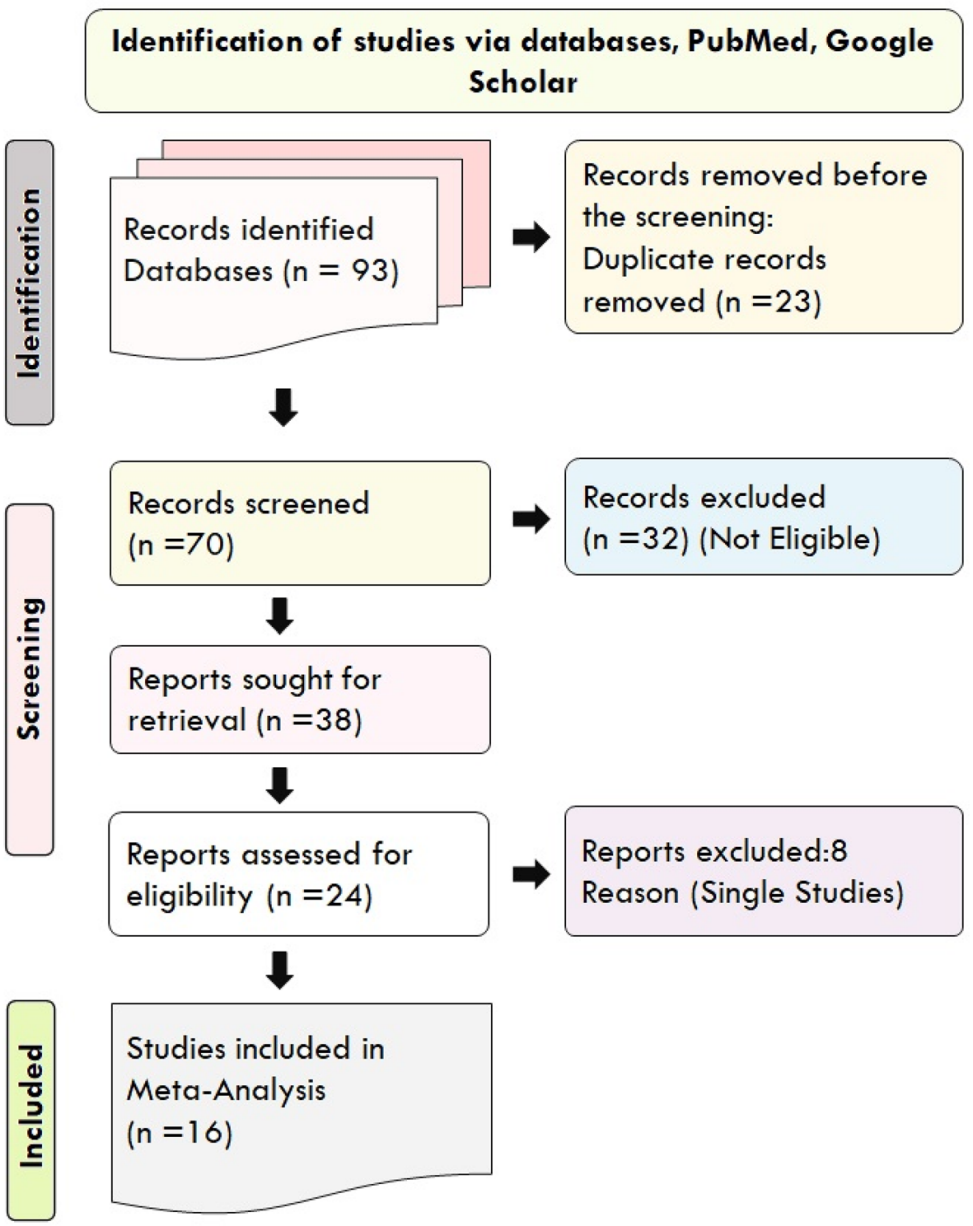
Selection of literature according to PRISMA (Preferred Reporting Items for Systematics Reviews and Meta-Analysis) guidelines.

**Figure 2:**
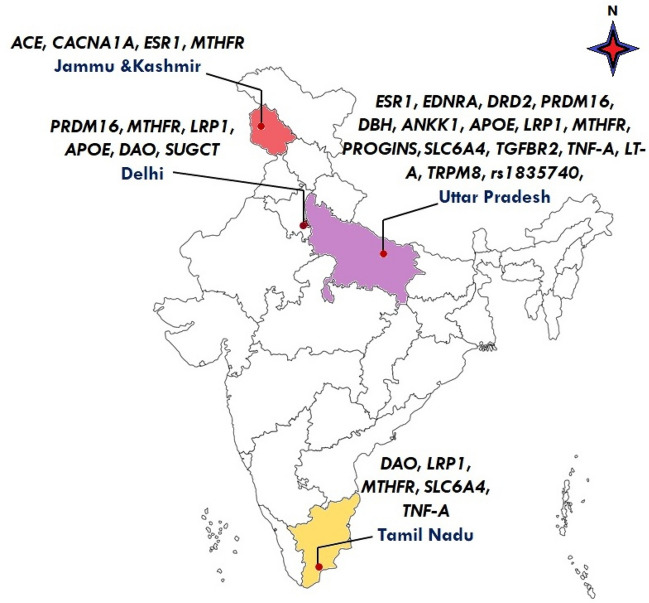
Indian Map representing different genes explored in 3 different states of India population.

Using the strategy of systematic way of literature survey (Fig. 1), a total of 24 studies was found (Table 1) which explored about 21 genes with 31 variants (Table 1) from 4 different states of India (3 states from north India and 1 state from south India) (Fig. 2) (Paint—Microsoft Apps). Only nine genes have been studied more than two times in the Indian population and thus were found eligible for the meta-analysis and these include six studies which have explored MTHFR gene^13, 16–18, 22, 23^, three studies for ACE (I/D polymorphism)^13, 17, 24^, LRP1- rs11172113^19, 26, 28^, PRDM16- rs2651899^19, 26^, TRPM8- rs10166942 and rs10504861^21, 26^, ESR1 PvuII and XbaI^15, 29, 37^, DAO- rs10156191, rs2052129^20, 35^ and TNF-α G308A^25, 30^.

### Study characteristics

After finding nine genes eligible for meta-analysis, the second step was the inclusion into the meta-analysis of eligible studies which were done based on the NOS (Table 2) and HWE criteria. If the NOS were six or more than it and if the control population were in HWE respectively the study was included After which two variants such as A1298C of *MTHFR*and rs10166942 of *TRPM8* were excluded due to not being found in HWE (Supplementary Tables 2 S1 and 5 S1).

**Table 2:** Newcastle–Ottawa Scale (NOS) for assessing the quality of non-randomized studies in meta-analyses^**†**^

Author/ Reference	Selection				Comparability	Exposure			Final Score
	Is the case definition adequate?	Representativeness of the cases	Selection of Controls	Definition of Controls	Comparability of cases and controls on the basis of the design or analysis	Ascertainment of exposure	Same method of ascertainment for cases and controls	Non-Response rate	
Aiswarya et al.^22^	**IHS (1)**	**Hospital (1)**	HB (0)	**Yes (1)**	**Yes (1)**	**Interview (1)**	**Yes (1)**	*NG (0)*	***6***
Ghosh et al.^25^	**IHS (1)**	**Hospital (1)**	HB (0)	**Yes (1)**	**Yes (1)**	**Interview (1)**	**Yes (1)**	*NG (0)*	***6***
Ghosh et al.^36^	**IHS (1)**	**Hospital (1)**	HB (0)	**Yes (1)**	**Yes (1)**	**Interview (1)**	**Yes (1)**	*NG (0)*	***6***
Ghosh et al.^26, 27^	**ICHD-2 (1)**	**Hospital (1)**	**PB (1)**	**Yes (1)**	*No (0)*	**Interview (1)**	**Yes (1)**	*NG (0)*	***6***
Ghosh et al.^26, 27^	**IHS (1)**	**Hospital (1)**	HB (0)	**Yes (1)**	*No (0)*	**Interview (1)**	**Yes (1)**	*NG (0)*	***5***
Jasrotia et al.^17^	**IHS (1)**	**Hospital (1)**	**PB (1)**	**Yes (1)**	*No (0)*	**Interview (1)**	**Yes (1)**	*NG (0)*	***6***
Joshi et al.^13, 14^	**IHS (1)**	**Hospital (1)**	HB (0)	**Yes (1)**	**Yes (1)**	**Interview (1)**	**Yes (1)**	*NG (0)*	***6***
Joshi et al.^13, 14^	**IHS (1)**	**Hospital (1)**	HB (0)	**Yes (1)**	**Yes (1)**	**Interview (1)**	**Yes (1)**	*NG (0)*	***6***
Joshi et al.^15^	**IHS (1)**	**Hospital (1)**	HB (0)	**Yes (1)**	**Yes (1)**	**Interview (1)**	**Yes (1)**	*NG (0)*	***6***
Joshi et al.^33^	**IHS (1)**	**Hospital (1)**	HB (0)	**Yes (1)**	**Yes (1)**	**Interview (1)**	**Yes (1)**	*NG (0)*	***6***
Kaur et al.^19, 21^	**IHS (1)**	**Hospital (1)**	HB (0)	**Yes (1)**	**Yes (1)**	**Interview (1)**	**Yes (1)**	*NG (0)*	***6***
Kaur et al.^19, 21^	**IHS (1)**	**Hospital (1)**	HB (0)	**Yes (1)**	**Yes (2)**	**Interview (1)**	**Yes (1)**	*NG (0)*	***7***
Kaur et al.^20^	**IHS (1)**	**Hospital (1)**	HB (0)	**Yes (1)**	**Yes (1)**	**Interview (1)**	**Yes (1)**	*NG (0)*	***6***
Kaur et al.^19, 21^	**IHS (1)**	**Hospital (1)**	**PB (1)**	**Yes (1)**	**Yes (1)**	**Interview (1)**	**Yes (1)**	*NG (0)*	***7***
Kesavan et al.^30^	**ICHD-3 (1)**	**Hospital (1)**	HB (0)	**Yes (1)**	*No (0)*	**Interview (1)**	**Yes (1)**	*NG (0)*	***5***
Kesavan et al.^34^	**IHS (1)**	**Hospital (1)**	**PB (1)**	**Yes (1)**	*No (0)*	**Interview (1)**	**Yes (1)**	*NG (0)*	***6***
Kumar et al.^29^	**ICHD-3 (1)**	**Hospital (1)**	HB (0)	**Yes (1)**	*No (0)*	**Interview (1)**	**Yes (1)**	*NG (0)*	***5***
Pandith et al.^16^	**IHS (1)**	**Hospital (1)**	HB (0)	**Yes (1)**	*No (0)*	**Interview (1)**	**Yes (1)**	*NG (0)*	***5***
Shoba et al.^28^	**IHS (1)**	**Hospital (1)**	HB (0)	**Yes (1)**	**Yes (1)**	**Interview (1)**	**Yes (1)**	*NG (0)*	***6***
Thomas et al.^35^	**IHS (1)**	**Hospital (1)**	HB (0)	**Yes (1)**	**Yes (1)**	**Interview (1)**	**Yes (1)**	*NG (0)*	***6***
Thomas et al.^35^	**IHS (1)**	**Hospital (1)**	HB (0)	**Yes (1)**	**Yes (1)**	**Interview (1)**	**Yes (1)**	*NG (0)*	***6***
Wani et al.^24^	**IHS (1)**	**Hospital (1)**	HB (0)	**Yes (1)**	**Yes (1)**	**Interview (1)**	**Yes (1)**	*NG (0)*	***6***

*HB* hospital based, *PB* population based, *HIS* InternationalHeadache Society, *ICHD-2/3* International Classification of Headache Disorders-2/3, *NG* not given.

Bold indicates positive point, Italics indicates no point, Bold italics indicates study included.

≥ 5 points/stars are consider as a good study and “ < 5 points” are considered as a high risk/biased study.

^†^Refer to Supplementary File.

### Meta-analysis

### *MTHFR-*C677T

In the present analysis, a total of 842 cases and 882 control subjects were included which were found after the inclusion of five studies representing four from the north Indian population^13, 16–18^ and one from the south Indian population^22^ and exclusion of one study due to not found in HWE^23^ (Supplementary Table 1 S1). The frequency of the risk allele was 0.195 (n = 329/1684) in contrast to the wild allele 0.804 (n = 1355/1684)within the case group while in a control group, the frequency of the risk allele was 0.168 (n = 297/1764) in contrast to wild allele i.e., 0.831 (n = 1467/1764) in the control group.

**Figure 3:**
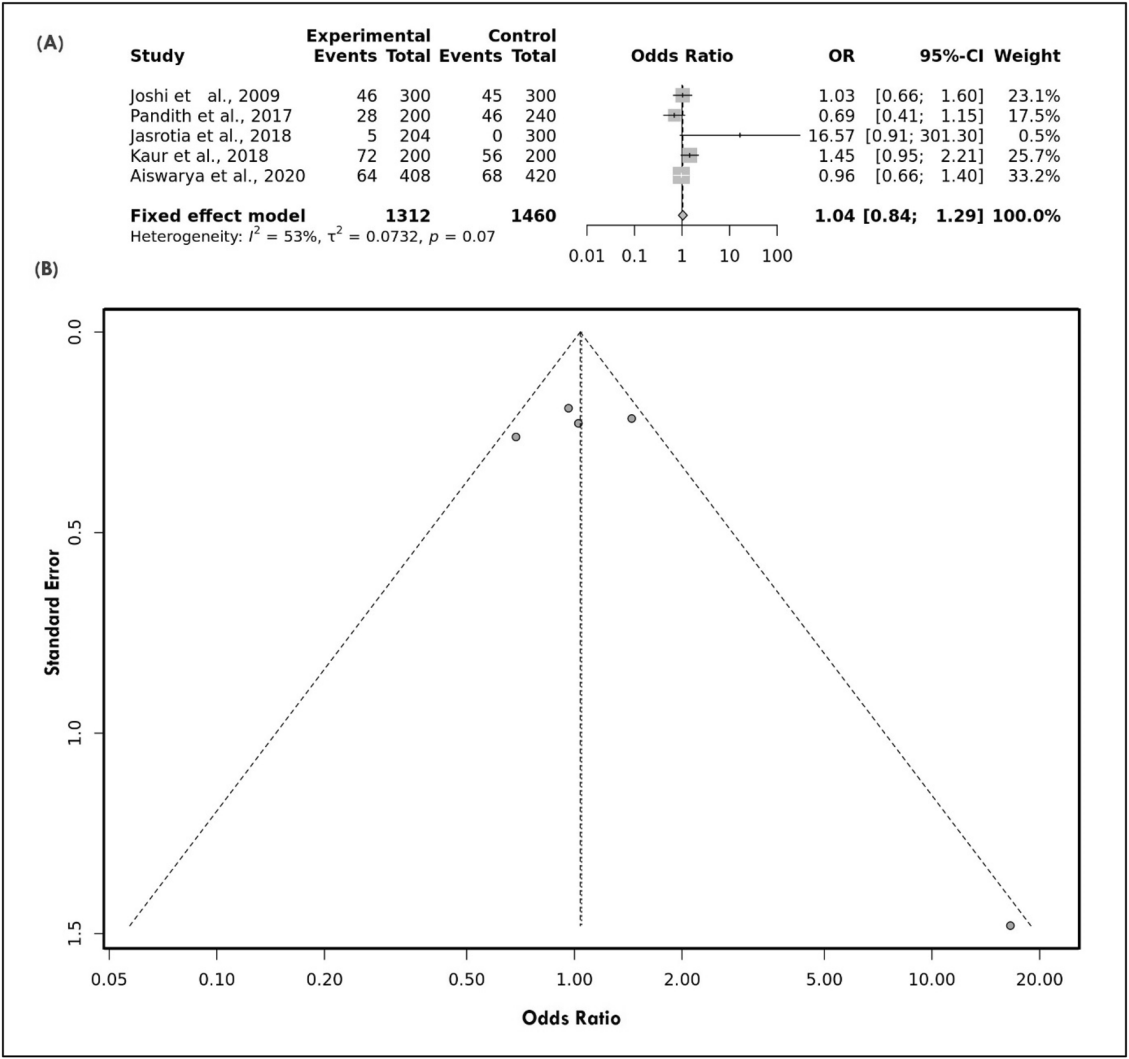
(**A**) Forest Plot of *MTHFR* allele model showing the non-significant association with the risk of overall migraine (**B**) Symmetrical Funnel Plot representing no publication bias.

To find out the association, a logistic regression model i.e., Odds Ratio associated with a 95% Confidence Interval, p-value < 0.05 were used. The present meta-analysis has shown that there was no significant association between the variant under study (C677T) and the risk of migraine in the Indian population after utilizing different genetic models and these include the allelic model (OR: 1.04 [0.84–1.29], I^2^ = 53%) (Fig. 3A), recessive model (OR: 1.31 [0.71–2.42], I^2^ = 38%), dominant model (OR: 1.08 [0.72–1.62], (I^2^ = 53%), and overdominant model (OR: 1.06 [0.70–1.60], (I^2^ = 53%). Subgrouping based on the criteria of the ”study conducted in which region of India i.e., such as South India (SI) and North India (NI)”, no significant association was observed (Supplementary Table 11 S1). After sub-grouping based on the clinical subtype i.e., MA and MWA, there was no significant association was found with any genetic models.

**Figure 4:**
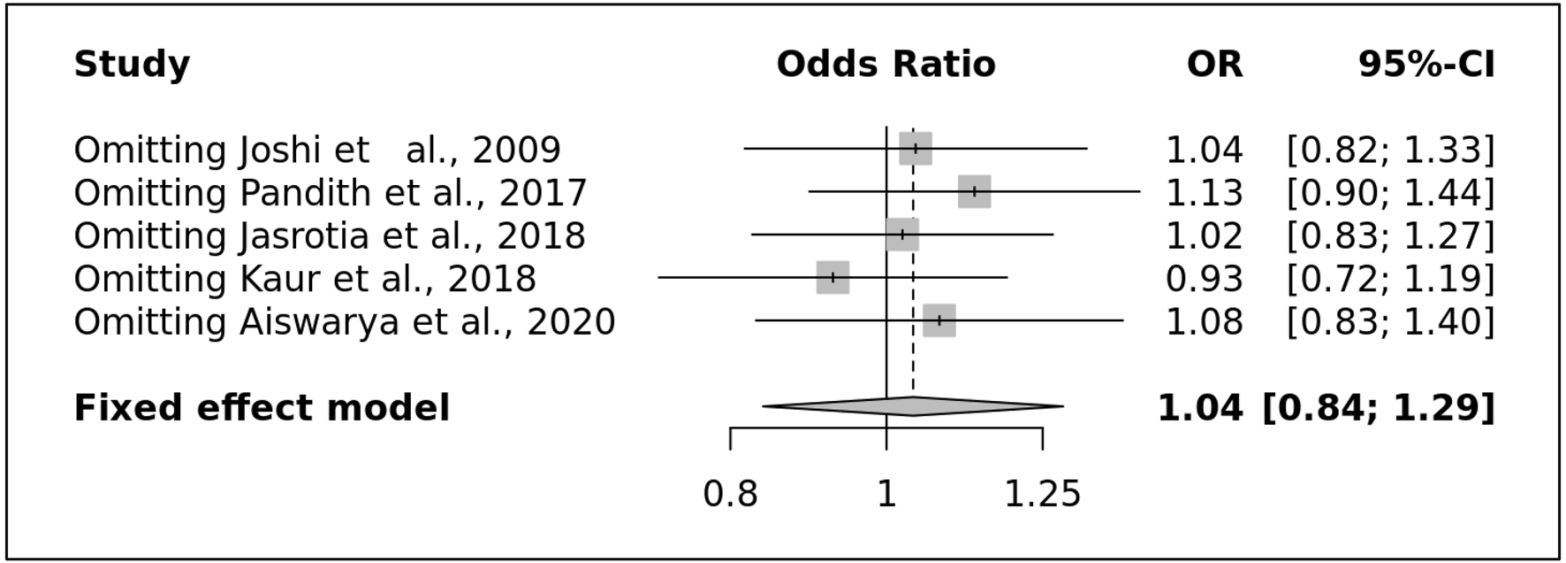
Sensitive plot representing allele model.

Egger's test, which is based on the connection between standard error and strength of association (log of OR), was used to examine publication bias across all studies included in the meta-analysis (p-value = 0.35). By placing the most accurate research on top and the least precise studies at the bottom of a scatter plot, we were able to create a "funnel plot" that displays the distribution of accuracy across all investigations. All genetic models resulted in symmetrical funnel plots, indicating that no publication bias existed. (Fig. 3B). The findings of a sensitive analysis performed on all genetic models by systematically removing individual studies showed that the pooled ORs were not significantly altered, confirming the excellent stability of the meta-analysis (Fig. 4).

### *ACE-*I/D

There are 3 studies^13, 17, 24^ (Supplementary Table 3 S1) that observed the frequency and association of polymorphism in the population of the Indian population. After pooling such independent studies, we found that the overall frequency of risk and wild allele was 0.410 (n = 289/704) and 0.589 (n = 415/704) in the patient group respectively. While in the control population, the frequency of the risk allele was considerably low i.e., 0.343 (n = 289/842) in comparison to the frequency of a minor allele in the patient group (q = 0.410).

**Figure 5:**
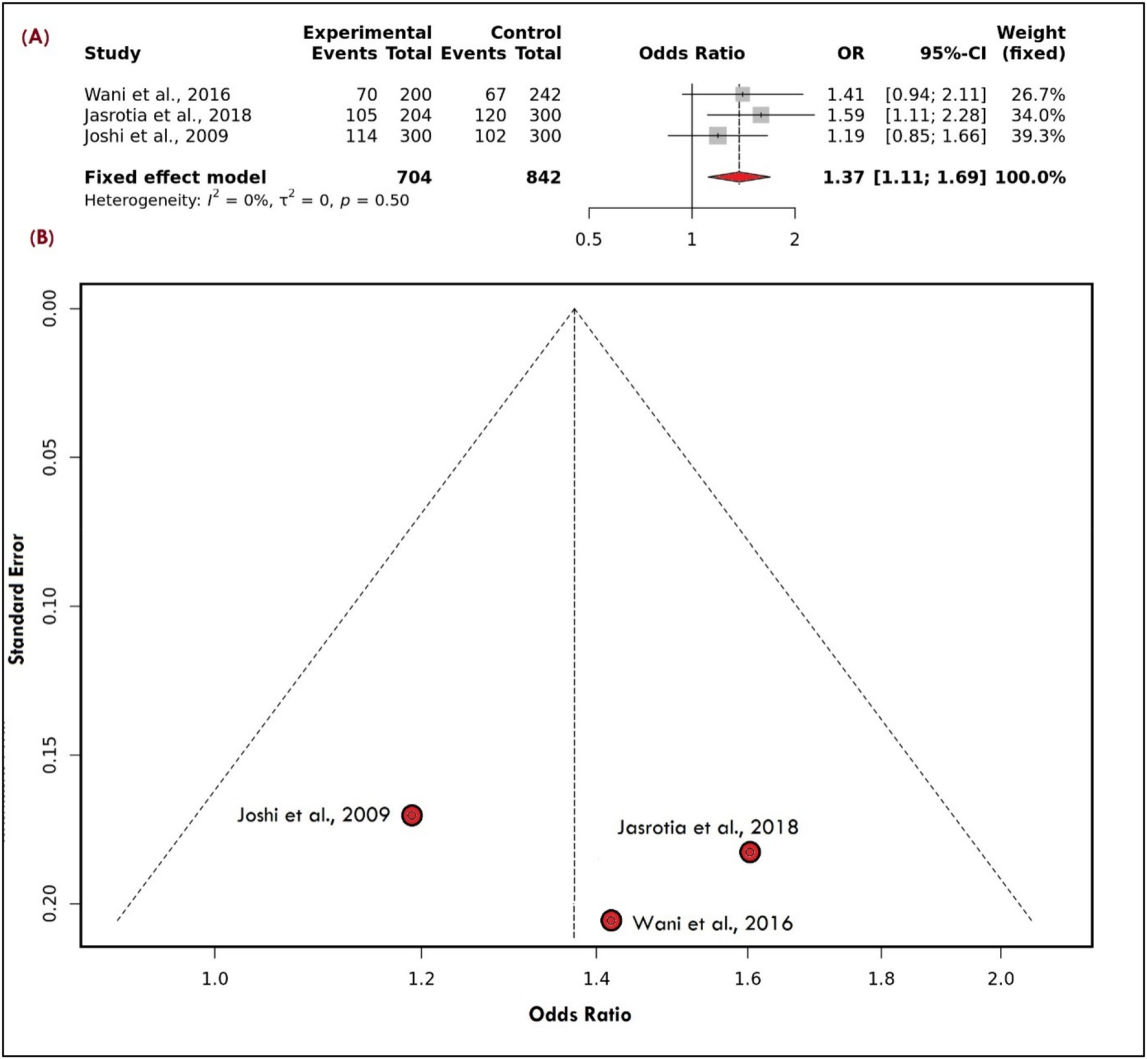
(**A**): Forest Plot of ACE allele model showing the significant association with the risk of overall migraine (**B**): Symmetrical Funnel Plot representing no publication bias.

The present meta-analysis found a significant association between the selected variant and risk of overall migraine after utilizing the allele (Fig. 5A) and recessive model (OR: 1.37 [1.11–1.69], I^2^ = 0%) and (OR: 2.05 [1.36–3.11], I^2^ = 0%) respectively in contrast to dominant and over-dominant model (OR: 1.29 [0.96–1.73], I^2^ = 0%) and (OR: 0.90 [0.67–1.19], I^2^ = 24%) respectively. After subgroup based on the clinical subtype i.e., MA and MWA, the variant showed a significant association after utilizing different genetic modes such as allele (OR: 1.41 [1.06–1.88], I^2^ = 0%), recessive (OR: 2.22 [1.29–3.83], I^2^ = 0%) in contrast to dominant (OR: 1.32 [0.88–1.98], I^2^ = 0%) and over-dominant model (OR: 0.90 [0.60–1.34], I^2^ = 39%) in MA group in compare to MWA where allele and recessive model showed significant association (OR: 1.33 [1.05–1.70], I^2^ = 42% and OR: 1.94 [1.21–3.11], I^2^ = 11%) in comparison to dominant and overdominant model (OR: 1.26 [0.89–1.77], I^2^ = 0% and OR: 0.90 [0.65–1.26], I^2^ = 0%) respectively. In addition, subgrouping based on the criteria of the ”study conducted in which region of India i.e., such as South India (SI) and North India (NI)”, was not done due to all studies were from north India.

**Figure 6:**
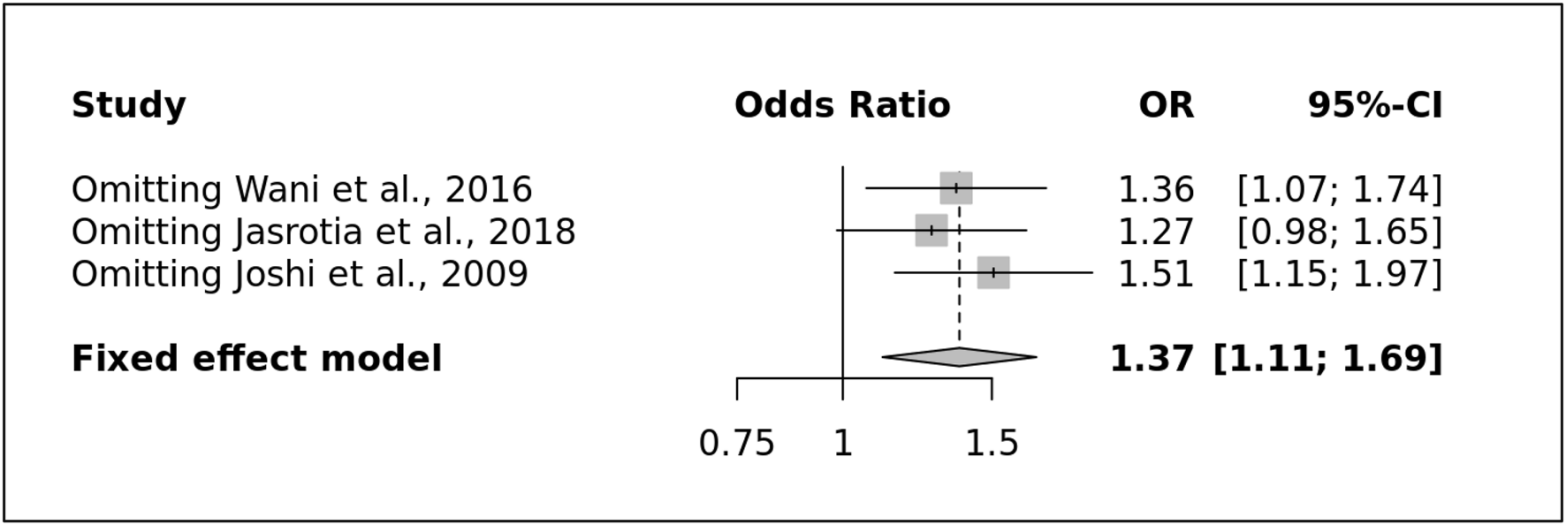
Sensitive plot representing allele model.

There was no evidence of publication bias because all funnel plots for genetic models were symmetrical (p-value = 0.68) (Fig. 5B) (Supplementary Table 14 S1). The good stability of the meta-analysis was confirmed by the results of a sensitive study done on all genetic models by carefully removing individual research (Fig. 6).

### ESR1

In the present study, we found two variants such as PvuII and XbaI of *ESR1* studies in the Indian population by three different research groups^14, 29, 37^ (Supplementary Tables 6 and 7 S1). Concerning ESR1-PvuII, a significant association has been found where the allele (OR: 1.47 [1.24–1.74], I^2^ = 0%), dominant (OR: 1.66 [1.30–2.12], I^2^ = 0%), and recessive model (OR: 1.91 [1.31–2.77], I^2^ = 0%) significantly increase the risk of migraine. After subgrouping based on clinical type criteria, a significant association was also found in both MA (Allele: OR: 1.72 [1.34–2.20], I^2^ = 0%, dominant: OR: 2.63 [1.75–3.96], I^2^ = 9%, overdominant: OR: 1.75 [1.23–2.50], I^2^ = 0% and recessive: OR: 1.79 [1.07–2.98], I^2^ = 0%), and MWA (allele: OR: 1.39 [1.15–1.67], I^2^ = 11%, dominant: OR: 1.43 [1.10–1.87], I^2^ = 55%), and recessive model: OR: 1.94 [1.30–2.89], I^2^ = 0%).

Concerning, after critical literature analysis, only two research publications were found^29, 37^ discussing the impact of XbaI polymorphism on the susceptibility of migraine and its type. The pooled OR of both studies did not show any significant association with the risk of migraine or with the migraine sub-type (Supplementary Table 12 S1). All genetic models resulted in symmetrical funnel plots, indicating that no publication bias existed. The findings of a sensitive analysis performed on all genetic models by systematically removing individual studies showed that the pooled ORs were not significantly altered, confirming the excellent stability of the meta-analysis.

### *TNF-α* G308A

After combining the two studies^25, 30^ (Supplementary Table 8 S1), there was a significant difference between the genotypic frequency in the patient group (GG: 77.80%, GA: 18.45%, & AA: 3.73%) in comparison to a control group (GG: 79.03%, GA: 18.43%, & AA: 2.53%). The frequency of risk allele (q) was found slightly more (q = 0.129) than the control group (q1 = 0.117).

When comparing the pooled results from the experimental (n = 856) and control (n = 868) groups, the association value was not statistically significant for any genetic model under study such as allelic (OR: 1.12 [0.84–1.51] I^2^ = 68: random model), dominant (OR: 1.08 [0.77–1.50]**,** I^2^ = 82%: random model), recessive (OR: 1.54 [0.70–3.39], I^2^ = 0.0%: fixed model), and over-dominant model (OR: 1.00 [0.70–1.42], I^2^ = 89%: random model) (Supplementary Table 15 S1). There was no subgrouping analysis in this variant since only one study investigated the clinical subtype^25^. All genetic models had symmetrical funnel plots, indicating that there was no publication bias. A sensitive investigation of all genetic models was also performed by removing each research one at a time. It was demonstrated that none of the pooled ORs were considerably influenced, indicating the meta-analysis findings' excellent stability.

### *LRP1*- rs11172113

**Figure 7:**
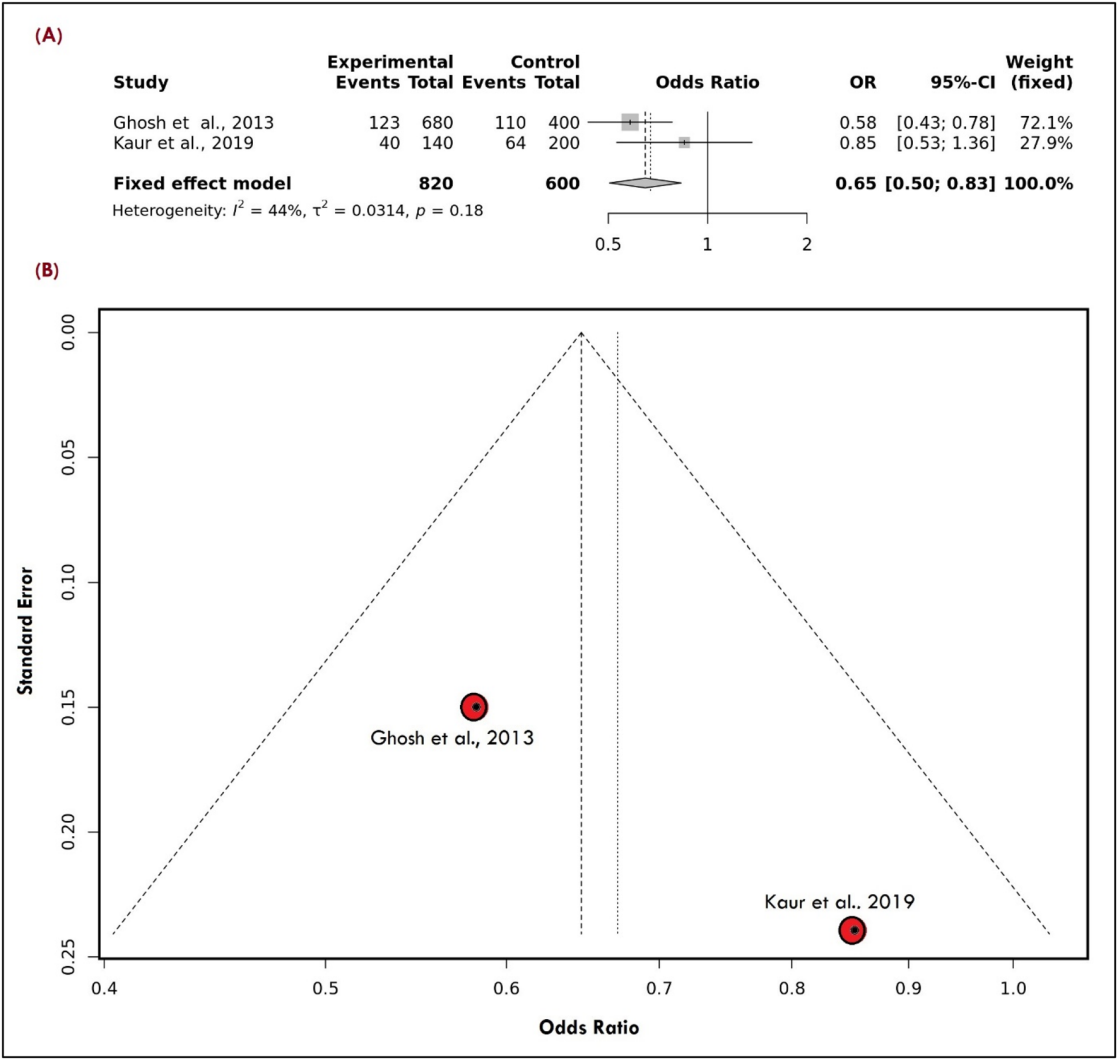
(**A**) LRP1 Allele showing the significant protective effect of a rare variant in the Indian population (**B**) Symmetrical Funnel Plot representing no publication bias.

In the present review, we found two studies^19, 26^ (Supplementary Table 9 S1) representing the north Indian population where the combined frequency of risk allele was less i.e., 0.198 (n = 163/410) in the patient's group in comparison to control group i.e., 0.29 (n = 174/600). To find out the risk using different genetic models, a protective role of variant (allelic model) (Fig. 7A) was observed with an OR of 0.65 [0.50–0.83] (I^2^ = 44%), dominant (OR: 0.48 [0.35–0.66], I^2^ = 12%), in contrast to recessive and over-dominant (OR: 1.29 [0.22–7.59], I^2^ = 91%) and (OR: 0.10 [0.00–5.43], I^2^ = 88%) respectively where the non-significant association was observed.

**Figure 8:**
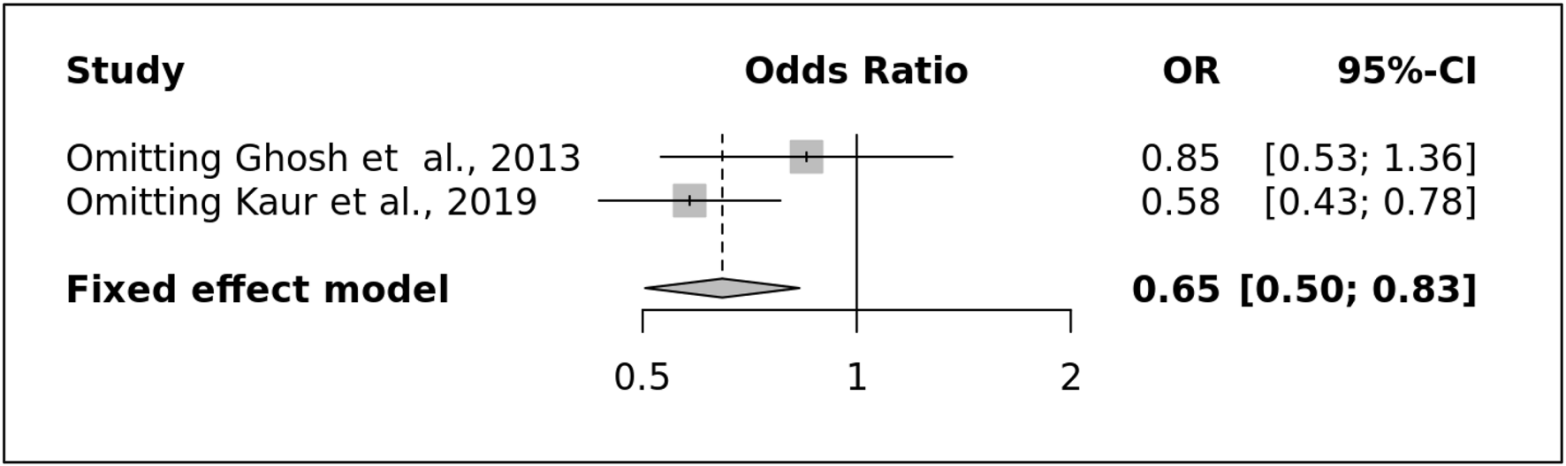
LRP1 migraine sensitivity plot for allele model.

After clinical sub-grouping of migraine, it was observed that allele (OR: 0.54 [0.37–0.78], I^2^ = 52%), dominant (OR: 0.47 [0.30–0.73], I^2^ = 0%), and over-dominant (OR: 0.54 [0.34–0.86], I^2^ = 0%) significantly showed protective role in MA. But, in the case of MWA, only dominant (OR: 0.68 [0.49–0.95], I^2^ = 58%) and over-dominant model (OR: 0.63 [0.45–0.88], I^2^ = 0%) showed a protective role in contrast to allele (OR: 0.88 [0.46–1.68], I^2^ = 82%) and recessive (OR: 1.12 [0.65–1.93], I^2^ = 74%) where a non-significant association was observed. There was no evidence of publication bias because all genetic models produced symmetrical funnel plots(Fig. 7B). The good stability of the meta-analysis was confirmed by the results of a sensitive study done on all genetic models by carefully deleting individual research(Fig. 8).

### DAO- rs10156191

After combining studies^20, 35^ (Supplementary Table 10 S1), it was observed that in the patient’s group, the heterozygote (CT: 29.42%) and homozygous recessive (TT: 5.71%) genotypes are slightly greater than the heterozygote (CT: 21.14%) and homozygous recessive (TT: 3.42%) genotype in control’s group. The frequency of the risk allele (q = 0.204) in the patient group was more than the frequency of the risk allele in the control group (q1 = 0.14).

The present meta-analysis provides pieces of evidence that allele (OR: 3.86 [0.37–39.98], I^2^ = 81%) and recessive model (OR: 1.47 [0.69–3.12], I^2^ = 52%) showed non-significant association with the risk of migraine in contrast to dominant (OR: 1.69 [1.19–2.42], I^2^ = 69%) and over-dominant model (OR: 1.62 [1.12–2.34], I^2^ = 13%) which significantly increase the risk of migraine in Indian population. All genetic models yielded symmetrical funnel plots, eliminating publication bias. A sensitive study on all genetic models carefully removing individual studies proved the meta-analysis's stability.

### *PRDM16*-rs2651899

Concerning *PRDM16*- rs2651899 (Supplementary Table 4 S1), the frequency of the risk allele in the case group was slightly higher i.e., 0.469 (n = 459/978) compared to risk allele frequency in the control group i.e., 0.471 (n = 330/700). In addition, no significant association was observed in migraine or any clinical subtype (MA and MWA) after utilizing any genetic models (Supplementary Table 13 S1).

### Trial sequential analysis

**Figure 9:**
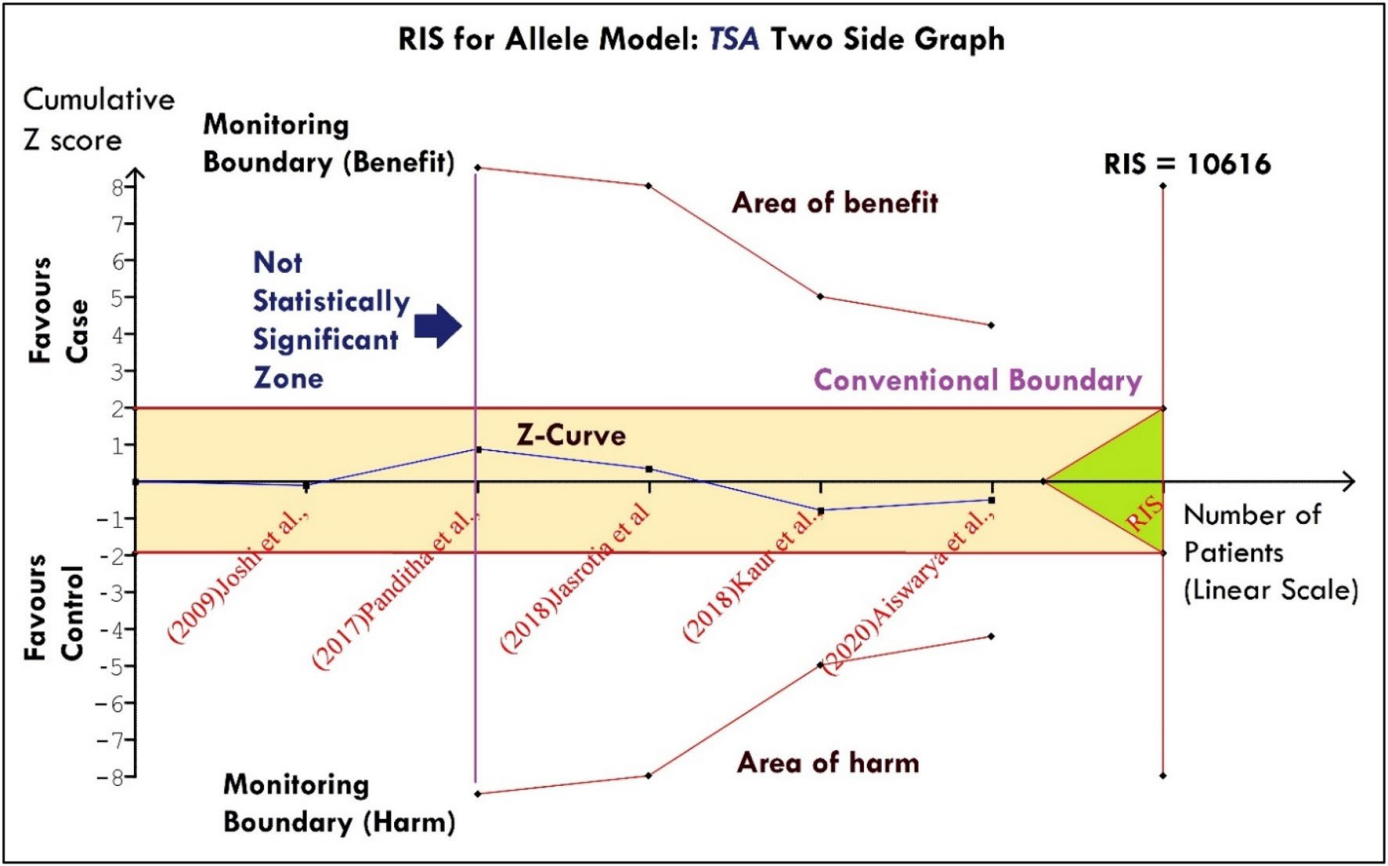
TSA graph for MTHFR-C677T (allele model) showed a non-significant result with less sample size/ power therefore required more studies to find out the association.

After finding a non-significant association for *MTHFR-*C677T, *DAO*- rs10156191, *TNF-α* G308A, and *ESR1*-XbaI, the required sample size estimation was done for allele model using TSA. For the *MTHFR-*C677T, the last point of the Z-curve reached or positioned within the conventional boundary which is considered as a statistically non-significant zone therefore, we cannot conclude that there is any risk association between the variant under study and diseases. Therefore, to achieve power (RIS: 10,616) further studies are required (Fig. 9). Concerning remaining *DAO*- rs10156191, *TNF-α* G308A, and *ESR1*-XbaI, the TSA showed “Boundary RIS is ignored due to little information use”.

### Protein–protein interaction

**Figure 10:**
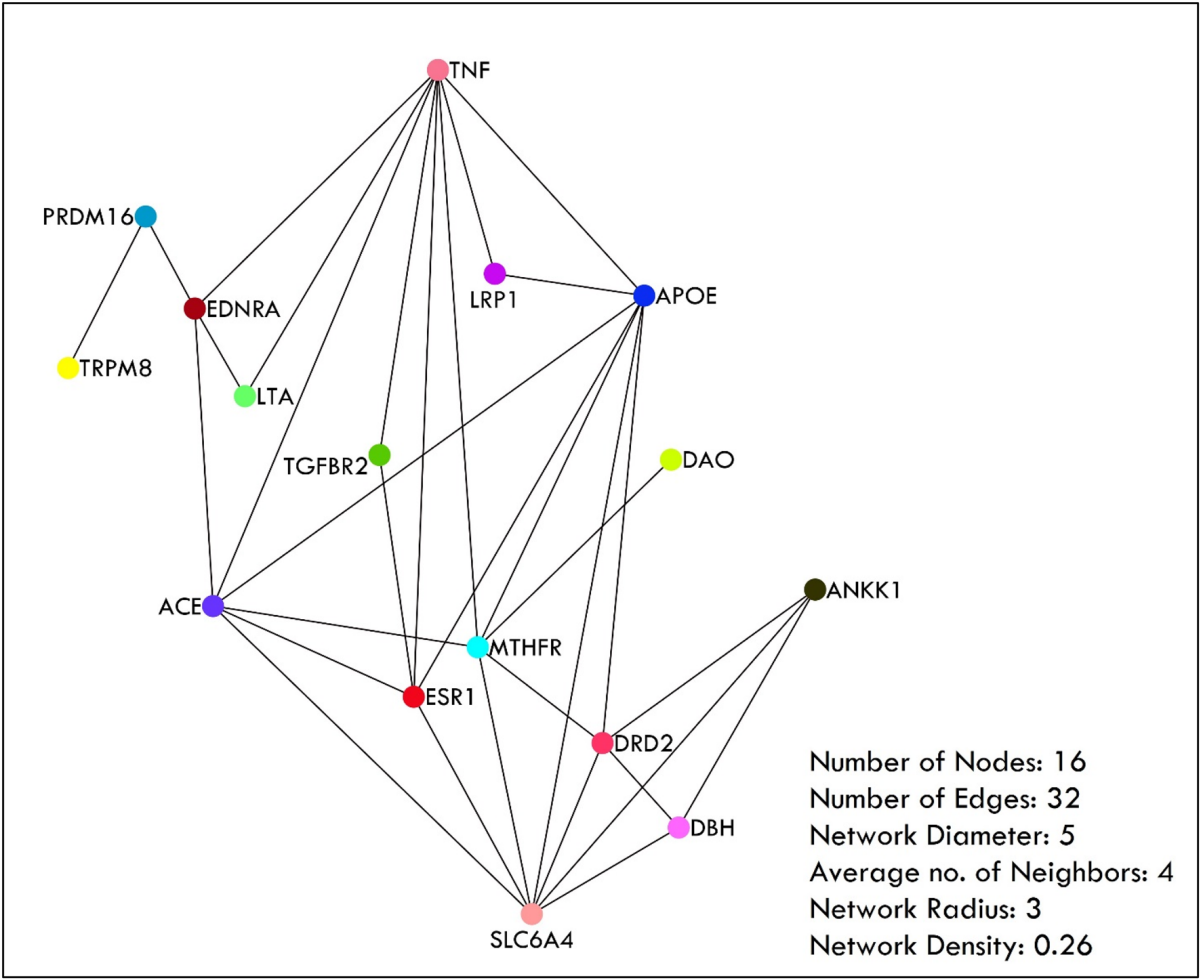
Protein–Protein interaction where the TNFA shows the highest node.

In the present study, we also aimed to find the most connected node in the list of genes studied in the population of India. Therefore, the String database which is a potential PPI tool that collects data from several online databases was used. Concerning the PPI setting, medium confidence (40%-69%), with active interaction sources which include test mining, experiments, databases, co-expression, neighborhood, gene fusion, and co-occurrence were utilized. We found that there were 32 connections/ edges between the processed 16 nodes/protein with an average node degree of four and six expected number of edges, 0.683 of average local clustering coefficient, and a significant PPI enrichment p-value (1.57e-14). It was observed that the highest degree (Degree: 8) was found with TNF-α followed by APOE and SLC6A4 (Degree: 7) (Table 3). String PPI was later edited and presented using Cytoscape tool version 3.9.1, which is an open-source bioinformatics software platform for visualizing molecular interaction networks and integrating them with gene expression profiles and other state data (Fig. 10).

**Table 3:** Protein–Protein Interaction.

Gene name/Node	Average shortest path length	Betweenness centrality	Closeness centrality	Clustering coefficient	Degree
TNF	1.666667	0.287755	0.6	0.321429	8
APOE	1.733333	0.111633	0.576923	0.52381	7
SLC6A4	1.8	0.199478	0.555556	0.47619	7
ACE	1.666667	0.179569	0.6	0.6	6
MTHFR	1.8	0.175918	0.555556	0.466667	6
ESR1	1.933333	0.053039	0.517241	0.6	5
DRD2	2.2	0.049887	0.454545	0.6	5
EDNRA	2	0.257007	0.5	0.333333	4
ANKK1	2.6	0	0.384615	1	3
DBH	2.6	0	0.384615	1	3
LRP1	2.266667	0	0.441176	1	2
PRDM16	2.8	0.133333	0.357143	0	2
LTA	2.4	0	0.416667	1	2
TGFBR2	2.333333	0	0.428571	1	2
DAO	2.733333	0	0.365854	0	1
TRPM8	3.733333	0	0.267857	0	1

## Discussion

Migraine is considered a complex disorder with polygenic inheritance and it has been shown by the most recent and updated meta-analysis of GWAS data has shown that numerous genes contribute to the risk of diseases with small effect sizes^7^. Other than the advanced GWAS, multiple studies have been conducted in different populations and found different genes. Specifying with the example of population, many genes have also been explored in the Indian population belonging to Asian ethnic groups constituted of different states (Fig. 2). Within the same population, association disparity was found in many genes and risk of migraine. Therefore, the present meta-analysis aimed to find out the precise risk between the different genes that have been explored in the past.

We have found that the variant ”C677T” of *MTHFR* showed a non-significant association with the risk of overall migraine in the Indian population which supports the independent studies^13, 16, 18^ in contrast to the positive association found by different independent studies^17, 22, 23^. Comparing the present pooled result with the most recent meta-analysis which discussed the association of C677T with the risk of migraine, showed a significant risk association^40^. However their selection of studies was before December 2018 and only two studies from the Indian population were included^13, 18^ and also missed the inclusion of one more study^17^. In addition to meta-analysis, we have also estimated the ”Required sample Size” using trial sequential analysis and observed that the Z-curve was unable to cross the required information/ sample size (Fig. 9). Therefore, we cannot conclude that there is any risk association between the variant under study and diseases and thus required more sample size.

Concerning the *ACE*-I/D polymorphism, the present meta-analysis showed a significant association between the variant of interest and the risk of migraine and both clinical subtypes such as MA and MWA. This present study supports the independent study by Jasrotia and group and Joshi and group^13, 17^ in contrast non-significant association was observed by Wani and group^24^. Interestingly all studies^13, 17, 24^ were from north India, thus disparity between such might be due to different sample sizes i.e., control and case subjects. Evidence from the meta-analysis published in 2016 powered with 7334 patients and 22,990 control showed no relationship between the ACE I/D polymorphism and any migraine but upon subgrouping based on the criteria of ethnicity, they observed a protective effect against migraine with aura and without aura at least in the Turkish population^41^.

Regarding *ESR1* and its variant studied in the Indian population, only PvuII showed a significant association with the risk of migraine including both of its clinical subtypes in contrast to XbaI. which was consistent with Kumar and group^29^ in contrast to Ghosh and group^37^. The other intronic variant i.e., PvuII which is separated by the 50 bp from XbaI, two studies discussed the association in the respective populations, and upon combing both studies, the present pooled meta-analysis showed significant association with the risk of migraine including both of its clinical subtypes which supports the result observed by Joshi and group^14^ in contrast to Kumar and group^29^.

TNF-alpha is known for its critical role in pro-inflammation and critical regulator of microglial activation which leads to the initiation of neurogenic-neuroinflammation^6^. But the presence of a functional variant i.e., − 308 G > A leads to elevated plasma level of protein thus hindering the susceptibility of inflammation threshold. In the present study, after analysis of two independent studies^25, 30^ we did not find any significant association after utilizing different genetic models and risk of migraine which was the opposite of what was observed by the included independent study^25, 30^. This prime reason for such disparity might be due to the different regions one is from north India^25^ and the other is from south India^30^. Therefore, it is very important to conduct more studies in the respective population to find out the precise result. Comparing our meta-analytic data with the pre-existing meta-analysis, the results were found consistent with different studies^30, 42–44^ in contrast to Chen and group^44^.

Concerning the *LRP1*- rs11172113, the present review observed a protective role of variant (allelic model) (Fig. 7A) with overall migraine, and such protective effect was found consistent with both of its clinical subtypes. Thus, the present pooled result supports the result that Ghosh and group previously observed^26^ in contrast to Kaur and group^19^. Each study was observed in the north Indian population, but such disparity might be due to the low sample size and also the patient group was not in HWE^19^. Comparing our meta-result with the overall pooled result presented by Siokas and the group where they observed a non-significant association between the rs11172113 and risk of migraine (OR: 1,10 [0.84–1.44], I^2^: 68%)^45^.

## Strengths and limitations of the present meta-analysis

The prime strength of the present pairwise meta-analysis is the strategy utilized for the literature survey, then the inclusion of searched studies based on the criteria discussed (Section ”Inclusion and exclusion features”), and secondly the use of statistical analysis for finding the risk association between the different risk variants and diseases under consideration. Thirdly, the presentation of pooled summary estimates is considerably simpler to understand. Fourth, we have also found the risk attribution between the selected variants and migraine subtypes (MA and MWA). Fifth, we also presented the protein–protein interaction in an attempt to find out the most connected node in the network of nodes selected from the population under study. Sixth, a precise risk attribution toward the risk of migraine within a specific population i.e., the Indian population has been established. Apart from the strength, the first limitation is that migraine is an extremely heterogeneous condition, as all studies have diagnosed the suspected individual using criteria of ICHD-3 / HIS, but still, there could be misclassification. Second, the present analysis is only limited to clinical subgrouping and no subgroup was done based on gender. Also, there was an incredible disparity in sample size between the studies. Also, the risk of non-significantly associated variants can be modified by different modifier genes which were not explored in the present study. Additionally, the risk of disease can be attributed to the interaction between the markers of the same gene. In addition, concerning with the included studies in the meta-analysis were not enough, therefore for a precise estimate, more studies are required.

## Future perspectives

In the present study, we aimed to find out the critical gene or genes that are responsible for the significant risk attribution toward disease susceptibility within a specific population (India) using a high statistical meta-analytical research approach. Different genes such as *ACE*-I/D, and *ESR1*-PvuII showed a significant association with the risk of migraine in contrast to *LRP1-*rs11172113 and *MTHFR*-C677T, *PRDM16*-rs2651899, *DAO*-rs10156191which showed protective and non-significant association in respect to Indian population respectively. We also noticed that there was much disparity in the sample size between studies, specifically the patient’s group was even not found in HWE. Also, the ratio between case and control was not even equal in the different studies, and for a fixed sample size, the chi-square test for independence is most powerful if the number of cases is equal to the number of controls (i.e., 1:1)^46^. Additionally, we can increase or recruit more controls to boost the study's statistical power if we are unable to find enough cases, but only up to 4 controls for every one case. Given the expense of recruiting them, adding more controls (more than four) might limit the increase in statistical power beyond this ratio^47^. There have only been a few, sometimes just two, studies exploring specific variants, which makes it necessary for more research to be done to support the risk attribution hypothesis.

## Conclusion

In conclusion, this present meta-analysis showed that the ACE-DD variant and *ESR1-*PvuII showed a significant risk of migraine in the Indian population in contrast to *LRP1-* rs11172113 which showed a protective role in the respective population.

### Supplementary Information

Supplementary Information.

## Data Availability

All data generated or analyzed during this study are included in this article. Further inquiries can be directed to the corresponding author.

## Abbreviations

ICHD-3: International classification of headache disorder 3rd edition
MA: Migraine aura
MWA: Migraine without aura
CSD: Cortical spreading depression
GWAS: Genome-wide association study
NCBI: National center for biotechnology information
MEDLINE: Medical literature analysis and retrieval system online
PRISMA: Preferred reporting items for systematics reviews and meta-analysis
IHS: International headache society
HWE: Hardy–Weinberg equilibrium
OR: Odds ratio
CI: Confidence interval
NOS: Newcastle–Ottawa quality assessment scale
I^2^: I Square
PPI: Protein–protein interaction
SI: South India
NI: North India
SNP: Single nucleotide polymorphism
SNV: Single nucleotide variation

## References

[1] Sudershan A, Mahajan K, Singh K, Dhar MK, Kumar P (2022). The complexities of migraine: A debate among migraine researchers: A review. Clin. Neurol. Neurosurg..

[2] Leao AAP (1944). Spreading depression of activity in the cerebral cortex. J. Neurophysiol..

[3] Bolay H, Reuter U, Dunn AK, Huang Z, Boas DA, Moskowitz MA (2002). Intrinsic brain activity triggers trigeminal meningeal afferents in a migraine model. Nat. Med..

[4] Ramachandran R (2018). Neurogenic inflammation and its role in migraine. Semin. Immunopathol..

[5] Spekker E, Laborc KF, Bohár Z, Nagy-Grócz G, Fejes-Szabó A, Szűcs M, Vécsei L, Párdutz Á (2021). Effect of dural inflammatory soup application on activation and sensitization markers in the caudal trigeminal nucleus of the rat and the modulatory effects of sumatriptan and kynurenic acid. J. Headache Pain.

[6] Sudershan A, Younis M, Sudershan S, Kumar P (2023). Migraine as an inflammatory disorder with microglial activation as a prime candidate. Neurol. Res..

[7] Hautakangas H, Winsvold BS, Ruotsalainen SE, Bjornsdottir G, Harder AVE, Kogelman LJA, Thomas LF, Noordam R, Benner C, Gormley P, Artto V, Banasik K, Bjornsdottir A, Boomsma DI, Brumpton BM, Burgdorf KS, Buring JE, Chalmer MA, de Boer I, Pirinen M (2022). Genome-wide analysis of 102,084 migraine cases identifies 123 risk loci and subtype-specific risk alleles. Nat. Genet..

[8] Kulkarni GB, Rao GN, Gururaj G, Stovner LJ, Steiner TJ (2015). Headache disorders and public ill-health in India: Prevalence estimates in Karnataka State. J. Headache Pain.

[9] Ray BK, Paul N, Hazra A, Das S, Ghosal MK, Misra AK, Banerjee TK, Chaudhuri A, Das SK (2017). Prevalence, burden, and risk factors of migraine: A community-based study from Eastern India. Neurol. India.

[10] Nandha R, Chhabra MK (2013). Prevalence and clinical characteristics of headache in dental students of a tertiary care teaching dental hospital in Northern India. Int. J. Basic Clin. Pharmacol..

[11] Sastry AS, Kumar A, Pathak A, Chaurasia RN, Singh VK, Joshi D, Singh V, Mishra VN (2022). The pattern of primary headache in the North India population: a hospital-based study. Int. J. Neurosci..

[12] Sudershan A, Pushap AC, Younis M, Sudershan S, Bhagat S, Kumar H, Panjalyia RK, Kumar P (2023). Neuroepidemiology study of headache in the region of Jammu of north Indian population: A cross-sectional study. Front. Neurol..

[13] Joshi G, Pradhan S, Mittal B (2009). Role of the ACE ID and MTHFR C677T polymorphisms in genetic susceptibility of migraine in a north Indian population. J. Neurol. Sci..

[14] Joshi G, Pradhan S, Mittal B (2009). Role of the oestrogen receptor (ESR1 PvuII and ESR1 325 C→ G) and progesterone receptor (PROGINS) polymorphisms in genetic susceptibility to migraine in a North Indian population. Cephalalgia.

[15] Joshi G, Pradhan S, Mittal B (2010). No direct association of serotonin transporter (STin2 VNTR) and receptor (HT 102T> C) gene variants in genetic susceptibility to migraine. Dis. Markers.

[16] Pandith AA, Wani IY, Qasim I, Shah ZA, Sheikh S (2017). Evaluation of risk related to MTHFR 677C> T gene polymorphism in migraine patients in Kashmiri population. Open J. Prev. Med..

[17] Jasrotia R, Raina JK, Sharma M, Panjaliya RK, Kundal BR, Kumar P (2018). Relationship of MTHFR and ACE gene variations with migraine susceptibility: A case-control study in the population of North India (Jammu). Biosci. Biotechnol. Res. Asia.

[18] Kaur S, Ali A, Pandey AK, Singh B (2018). Association of MTHFR gene polymorphisms with migraine in North Indian population. Neurol. Sci..

[19] Kaur S, Ali A, Ahmad U, Pandey AK, Singh B (2019). rs2651899 variant is associated with risk for migraine without aura from North Indian population. Mol. Biol. Rep..

[20] Kaur S, Ali A, Siahbalaei Y, Ahmad U, Nargis F, Pandey AK, Singh B (2020). Association of Diamine oxidase (DAO) variants with the risk for migraine from North Indian population. Meta Gene.

[21] Kaur S, Ali A, Siahbalaei Y, Ahmad U, Pandey AK, Singh B (2019). Could rs4379368 be a genetic marker for North Indian migraine patients with aura?: Preliminary evidence by a replication study. Neurosci. Lett..

[22] Aiswarya PS, Husain RA, Kesavan P, Subramaniyan K, Ahmed SS, Ramakrishnan V (2020). Association of rs1801133 polymorphism with migraine susceptibility: A case-control study followed by updated meta-analysis and trial sequential analysis. Gene Rep..

[23] Thomas ASS, Saraswathy R, Thayanithy M (2022). Association of MTHFR (C677T and A1298C) gene variants polymorphisms with migraineurs: A case-control study. Appl. Sci. Eng. Progress.

[24] Wani IY, Sheikh S, Shah ZA, Pandith AA, Wani M, Asimi R, Wani M, Sheikh S, Mehraj I (2016). Association of ACE Gene I/D polymorphism with migraine in Kashmiri population. Ann. Indian Acad. Neurol..

[25] Ghosh J, Joshi G, Pradhan S, Mittal B (2010). Investigation of TNFA 308G> A and TNFB 252G> A polymorphisms in genetic susceptibility to migraine. J. Neurol..

[26] Ghosh J, Pradhan S, Mittal B (2013). Genome-wide-associated variants in migraine susceptibility: A replication study from North India. Headache J. Head Face Pain.

[27] Ghosh J, Pradhan S, Mittal B (2013). Identification of a novel ANKK1 and other dopaminergic (DRD2 and DBH) gene variants in migraine susceptibility. Neuromol. Med..

[28] Shoba US, Srinivasan G, Gundlapally J, Kuppamuthu K (2020). Association of Single Nucleotide Polymorphism rs11172113 of LRP1 Gene with Migraine in South Indian Population–A Study. Helix Rev. Bimonth. Int. J..

[29] Kumar S, Raina JK, Sudershan A, Mahajan K, Jasrotia R, Maharana C, Kumar P (2023). An association study of ESR1–XbaI and PvuII gene polymorphism in migraine susceptibility in the Jammu region. Eur. Neurol..

[30] Kesavan P, Satheesh AP, Husain RSRA, Veerappan U, Kannaian S, Ahmed SS, Veerabathiran R (2021). Genetic predisposition of TNFα gene polymorphism in South-Indian Migraineurs and meta-analysis. Front. Biosci.-Elite.

[31] Bashir A, Saleem S, Wani M, Rasool R, Wani IY, Gulnar A, Verma S (2014). Association of single nucleotide polymorphisms of CACNA1A gene in migraine. Indian J. Hum. Genet..

[32] Gupta R, Kumar V, Luthra K, Banerjee B, Bhatia MS (2009). Polymorphism in apolipoprotein E among migraineurs and tension-type headache subjects. J. Headache Pain.

[33] Joshi G, Pradhan S, Mittal B (2011). Vascular gene polymorphisms (EDNRA-231 G> A and APOE HhaI) and risk for migraine. DNA Cell Biol..

[34] Kesavan P, Satheesh AP, Rasheed AH, Veerappan U, Kannaian S, Veerabathiran R (2023). Association analysis of serotonin transporter gene polymorphism among the South-Indian migraineurs. Curr. J. Neurol..

[35] Thomas ASS, Saraswathy R, Anne A, Thayanithy M (2022). Association of serum copper levels and amine oxidase copper gene 1 (AOC1) with migraineurs. Appl. Sci. Eng. Progress.

[36] Ghosh J, Pradhan S, Mittal B (2011). Role of dopaminergic gene polymorphisms (DBH 19 bp indel and DRD2 Nco I) in genetic susceptibility to migraine in North Indian population. Pain Med..

[37] Ghosh J, Joshi G, Pradhan S, Mittal B (2012). Potential role of aromatase over estrogen receptor gene polymorphisms in migraine susceptibility: A case control study from North India. PloS One.

[38] Page MJ, McKenzie JE, Bossuyt PM, Boutron I, Hoffmann TC, Mulrow CD, Shamseer L, Tetzlaff JM, Akl EA, Brennan SE, Chou R, Glanville J, Grimshaw JM, Hróbjartsson A, Lalu MM, Li T, Loder EW, Mayo-Wilson E, McDonald S, McGuinness LA, Moher D (2021). The PRISMA 2020 statement: an updated guideline for reporting systematic reviews. BMJ Clin. Res..

[39] Wray NR, Wijmenga C, Sullivan PF, Yang J, Visscher PM (2018). Common disease is more complex than implied by the core gene omnigenic model. Cell.

[40] Rai V, Kumar P (2022). Relation between methylenetetrahydrofolate reductase polymorphisms (C677T and A1298C) and migraine susceptibility. Indian J. Clin. Biochem..

[41] Wan D, Wang C, Zhang X, Tang W, Chen M, Dong Z, Yu S (2016). Association between angiotensin-converting enzyme insertion/deletion polymorphism and migraine: A meta-analysis. Int. J. Neurosci..

[42] Gu L, Yan Y, Long J, Su L, Hu Y, Chen Q, Xie J, Wu G (2012). The *TNF-α*-308G/A polymorphism is associated with migraine risk: A meta-analysis. Experim. Ther. Med..

[43] Schurks M, Rist PM, Zee RY, Chasman DI, Kurth T (2011). Tumour necrosis factor gene polymorphisms and migraine: A systematic review and meta-analysis. Cephalalgia Int. J. Headache.

[44] Chen M, Tang W, Hou L, Liu R, Dong Z, Han X, Zhang X, Wan D, Yu S (2015). Tumor necrosis factor (TNF) -308G>A, nitric oxide synthase 3 (NOS3) +894G>T polymorphisms and migraine risk: A meta-analysis. PloS One.

[45] Siokas V, Liampas I, Aloizou AM, Papasavva M, Bakirtzis C, Lavdas E, Liakos P, Drakoulis N, Bogdanos DP, Dardiotis E (2022). Deciphering the role of the rs2651899, rs10166942, and rs11172113 polymorphisms in migraine: A meta-analysis. Medicina (Kaunas, Lithuania).

[46] Jewell NP (2003). Statistics for Epidemiology.

[47] Setia MS (2016). Methodology series module 2: Case-control studies. Indian J. Dermatol..

