# Supplementary material for: Comprehensive analysis of genes associated with migraine in the Indian population: a meta-analysis of genetic association studies with trial sequential analysis
Source: Sci Rep. 2023 Nov 4;13:19070. doi: 10.1038/s41598-023-45531-3 (PMC10625547; doi:10.1038/s41598-023-45531-3)
Supplement: Supplementary file 1 — Supplementary Information. [file 41598_2023_45531_MOESM1_ESM.docx]

**Supplementary File**

**Comprehensive Analysis of Genes Associated with Migraine in the Indian Population: A Meta-Analysis of Genetic Association Studies with Trial Sequential Analysis**

**Amrit Sudershan^1,2^, Agar Chander Pushap^3^, Meenakshi Bhagat^4^, Isha Sharma^4^, Hardeep Kumar^5^, Sanjeev K Digra^6,*^, Parvinder Kumar^1,4^***

1. Institute of Human Genetics, University of Jammu, Jammu, Jammu & Kashmir, India-180006
2. Department of Human Genetics, Sri Pratap College, Cluster University of Srinagar, Kashmir, Jammu & Kashmir, India-
3. Department of Education, Dakshina Bharat Hindi Prachar Sabha, Madras, India-600017
4. Department of Zoology, University of Jammu, Jammu, Jammu and Kashmir, India-180006
5. Department of Neurology, Super Specialty Hospital, Jammu, Jammu and Kashmir, India-180006
6. Department of Paediatrics, Sri Maharaja Gulab Singh Hospital, Government Medical College, Jammu, Jammu & Kashmir, India

*Corresponding author:

**Dr. Parvinder Kumar**

Sr. Prof & Deputy Coordinator

Department of Zoology &

Institute of Human Genetics,

University of Jammu, Jammu

Jammu & Kashmir, India-180006

**E-mail:**[drparvinderkumar@jammuuniversity.ac.in](mailto:drparvinderkumar@jammuuniversity.ac.in)

**&**[parvinderkb2003@gmail.com](mailto:parvinderkb2003@gmail.com)

**ORCID:** 0000-0003-1039-6638

**Prof (Dr.) Sanjeev K Digra**

Department of Paediatrics,

Sri Maharaja Gulab Singh Hospital,

Government Medical College, Jammu,

Jammu & Kashmir, India-180006

**Email:**[sanjeevahsaas@yahoo.co.in](mailto:sanjeevahsaas@yahoo.co.in)

**Supplementary Prisma Check List S1:PRISMA**

| **Section and Topic** | **Item #** | **Checklist item** | **Location where item is reported** |
| --- | --- | --- | --- |
| **TITLE** | | |  |
| Title | 1 | Identify the report as a systematic review. | Heading |
| **ABSTRACT** | | |  |
| Abstract | 2 | See the PRISMA 2020 for Abstracts checklist. | Checked |
| **INTRODUCTION** | | |  |
| Rationale | 3 | Describe the rationale for the review in the context of existing knowledge. | Page no. 3 |
| Objectives | 4 | Provide an explicit statement of the objective(s) or question(s) the review addresses. | Page no. 3 |
| **METHODS** | | |  |
| Eligibility criteria | 5 | Specify the inclusion and exclusion criteria for the review and how studies were grouped for the syntheses. | Page no. 4 |
| Information sources | 6 | Specify all databases, registers, websites, organisations, reference lists and other sources searched or consulted to identify studies. Specify the date when each source was last searched or consulted. | Page no. 4 |
| Search strategy | 7 | Present the full search strategies for all databases, registers and websites, including any filters and limits used. | Section 2.1 |
| Selection process | 8 | Specify the methods used to decide whether a study met the inclusion criteria of the review, including how many reviewers screened each record and each report retrieved, whether they worked independently, and if applicable, details of automation tools used in the process. | Page no. 5 |
| Data collection process | 9 | Specify the methods used to collect data from reports, including how many reviewers collected data from each report, whether they worked independently, any processes for obtaining or confirming data from study investigators, and if applicable, details of automation tools used in the process. | Page no. 5 |
| Data items | 10a | List and define all outcomes for which data were sought. Specify whether all results that were compatible with each outcome domain in each study were sought (e.g. for all measures, time points, analyses), and if not, the methods used to decide which results to collect. | SF Table 1-10 |
|  | 10b | List and define all other variables for which data were sought (e.g. participant and intervention characteristics, funding sources). Describe any assumptions made about any missing or unclear information. | Table 1 |
| Study risk of bias assessment | 11 | Specify the methods used to assess risk of bias in the included studies, including details of the tool(s) used, how many reviewers assessed each study and whether they worked independently, and if applicable, details of automation tools used in the process. | Forest Plot  Picture File |
| Effect measures | 12 | Specify for each outcome the effect measure(s) (e.g. risk ratio, mean difference) used in the synthesis or presentation of results. | Odds Ratio {95% CI] p-value <0.05 |
| Synthesis methods | 13a | Describe the processes used to decide which studies were eligible for each synthesis (e.g. tabulating the study intervention characteristics and comparing against the planned groups for each synthesis (item #5)). | Table 1-10 (SF) |
|  | 13b | Describe any methods required to prepare the data for presentation or synthesis, such as handling of missing summary statistics, or data conversions. | Page no. 5 |
|  | 13c | Describe any methods used to tabulate or visually display results of individual studies and syntheses. |  |
|  | 13d | Describe any methods used to synthesize results and provide a rationale for the choice(s). If meta-analysis was performed, describe the model(s), method(s) to identify the presence and extent of statistical heterogeneity, and software package(s) used. | Page no. 5-6 |
|  | 13e | Describe any methods used to explore possible causes of heterogeneity among study results (e.g. subgroup analysis, meta-regression). |  |
|  | 13f | Describe any sensitivity analyses conducted to assess robustness of the synthesized results. |  |
| Reporting bias assessment | 14 | Describe any methods used to assess risk of bias due to missing results in a synthesis (arising from reporting biases). |  |
| Certainty assessment | 15 | Describe any methods used to assess certainty (or confidence) in the body of evidence for an outcome. | 95% CI |
| **RESULTS** | | |  |
| Study selection | 16a | Describe the results of the search and selection process, from the number of records identified in the search to the number of studies included in the review, ideally using a flow diagram. | Figure 1 |
|  | 16b | Cite studies that might appear to meet the inclusion criteria, but which were excluded, and explain why they were excluded. | Section 2.2 |
| Study characteristics | 17 | Cite each included study and present its characteristics. |  |
| Risk of bias in studies | 18 | Present assessments of risk of bias for each included study. |  |
| Results of individual studies | 19 | For all outcomes, present, for each study: (a) summary statistics for each group (where appropriate) and (b) an effect estimates and its precision (e.g. confidence/credible interval), ideally using structured tables or plots. | Result section |
| Results of syntheses | 20a | For each synthesis, briefly summarise the characteristics and risk of bias among contributing studies. | - |
|  | 20b | Present results of all statistical syntheses conducted. If meta-analysis was done, present for each the summary estimate and its precision (e.g. confidence/credible interval) and measures of statistical heterogeneity. If comparing groups, describe the direction of the effect. | Done |
|  | 20c | Present results of all investigations of possible causes of heterogeneity among study results. | Test of Heterogeneity |
|  | 20d | Present results of all sensitivity analyses conducted to assess the robustness of the synthesized results. | Conducted |
| Reporting biases | 21 | Present assessments of risk of bias due to missing results (arising from reporting biases) for each synthesis assessed. | NA |
| Certainty of evidence | 22 | Present assessments of certainty (or confidence) in the body of evidence for each outcome assessed. | 95 CI |
| **DISCUSSION** | | |  |
| Discussion | 23a | Provide a general interpretation of the results in the context of other evidence. | Section 4 |
|  | 23b | Discuss any limitations of the evidence included in the review. | Page no 15 |
|  | 23c | Discuss any limitations of the review processes used. | Page no.15 |
|  | 23d | Discuss implications of the results for practice, policy, and future research. | Page no. 15-16 |
| **OTHER INFORMATION** | | |  |
| Registration and protocol | 24a | Provide registration information for the review, including register name and registration number, or state that the review was not registered. | NA |
|  | 24b | Indicate where the review protocol can be accessed, or state that a protocol was not prepared. | NA |
|  | 24c | Describe and explain any amendments to information provided at registration or in the protocol. | NA |
| Support | 25 | Describe sources of financial or non-financial support for the review, and the role of the funders or sponsors in the review. | Page no. 18 |
| Competing interests | 26 | Declare any competing interests of review authors. | Page no. 18 |
| Availability of data, code and other materials | 27 | Report which of the following are publicly available and where they can be found: template data collection forms; data extracted from included studies; data used for all analyses; analytic code; any other materials used in the review. | Supplementary file |

*From:*  Page MJ, McKenzie JE, Bossuyt PM, Boutron I, Hoffmann TC, Mulrow CD, et al. The PRISMA 2020 statement: an updated guideline for reporting systematic reviews.BMJ 2021;372:n71. doi: 10.1136/bmj.n71

For more information, visit:<http://www.prisma-statement.org/>

| **Section and Topic** | **Item #** | **Checklist item** | **Reported (Yes/No)** |
| --- | --- | --- | --- |
| **TITLE** | | |  |
| Title | 1 | Identify the report as a systematic review. | Yes |
| **BACKGROUND** | | |  |
| Objectives | 2 | Provide an explicit statement of the main objective(s) or question(s) the review addresses. | Yes |
| **METHODS** | | |  |
| Eligibility criteria | 3 | Specify the inclusion and exclusion criteria for the review. | Yes |
| Information sources | 4 | Specify the information sources (e.g. databases, registers) used to identify studies and the date when each was last searched. | Yes |
| Risk of bias | 5 | Specify the methods used to assess risk of bias in the included studies. | Yes |
| Synthesis of results | 6 | Specify the methods used to present and synthesise results. | Yes |
| **RESULTS** | | |  |
| Included studies | 7 | Give the total number of included studies and participants and summarise relevant characteristics of studies. | Yes |
| Synthesis of results | 8 | Present results for main outcomes, preferably indicating the number of included studies and participants for each. If meta-analysis was done, report the summary estimate and confidence/credible interval. If comparing groups, indicate the direction of the effect (i.e. which group is favoured). | Yes |
| **DISCUSSION** | | |  |
| Limitations of evidence | 9 | Provide a brief summary of the limitations of the evidence included in the review (e.g. study risk of bias, inconsistency and imprecision). | No |
| Interpretation | 10 | Provide a general interpretation of the results and important implications. | Yes |
| **OTHER** | | |  |
| Funding | 11 | Specify the primary source of funding for the review. | No |
| Registration | 12 | Provide the register name and registration number. | NO |

*From:*  Page MJ, McKenzie JE, Bossuyt PM, Boutron I, Hoffmann TC, Mulrow CD, et al. The PRISMA 2020 statement: an updated guideline for reporting systematic reviews. BMJ 2021;372:n71. doi: 10.1136/bmj.n71

For more information, visit:<http://www.prisma-statement.org/>

**SF1 (NOS): NEWCASTLE - OTTAWA QUALITY ASSESSMENT SCALE**

**CASE-CONTROL STUDIES**

Note: A study can be awarded a maximum of one star for each numbered item within the Selection and Exposure categories. A maximum of two stars can be given for Comparability.

**Selection**

1) Is the case definition adequate?

a) yes, with independent validation ** (1)**

b) yes, eg record linkage or based on self-reports

c) no description

2) Representativeness of the cases

a) consecutive or obviously representative series of cases ** (1)**

b) potential for selection biases or not stated

3) Selection of Controls

a) community controls ** (1)**

b) hospital controls

c) no description

4) Definition of Controls

a) no history of disease (endpoint) ** (1)**

b) no description of source

**Comparability**

1) Comparability of cases and controls on the basis of the design or analysis

a) study controls for _______________ (Select the most important factor.) ** (1)**

b) study controls for any additional factor **** (This criterion could be modified to indicate specific control for a second important factor.) **(1)**

**Exposure**

1) Ascertainment of exposure

a) secure record (eg surgical records) ** (1)**

b) structured interview where blind to case/control status ** (1)**

c) interview not blinded to case/control status

d) written self-report or medical record only

e) no description

2) Same method of ascertainment for cases and controls

a) yes ****

b) no

3) Non-Response rate

a) same rate for both groups ****

b) non respondents described

c) rate different and no designation

(<https://www.ohri.ca/programs/clinical_epidemiology/oxford.asp>)

**Supplementary table-1 S1:**Detail studies features exploring the MTHFR-C677T and risk of migraine and its clinical subtype

| **Study** | **Ethnicity** | **Control** | **Tech** | **Type** |  | **Case** |  |  | **Control** |  | **HWE.P-value** |
| --- | --- | --- | --- | --- | --- | --- | --- | --- | --- | --- | --- |
|  |  |  |  |  | **HW** | **HT** | **HR** | **HW** | **HT** | **HR** |  |
| Joshi et al., 2009 | Asian | HB | PCR-RFLP | Any Migraine | 104 | 46 | 0 | 108 | 39 | 3 | 0.97 |
|  |  |  |  | MA | 67 | 21 | 0 |  |  |  |  |
|  |  |  |  | MWA | 83 | 58 | 25 |  |  |  |  |
| Pandith et al., 2017 | Asian | HB | PCR-RFLP | Any Migraine | 73 | 26 | 1 | 80 | 34 | 6 | 0.69 |
|  |  |  |  | MA | 35 | 12 | 0 |  |  |  |  |
|  |  |  |  | MWA | 37 | 15 | 1 |  |  |  |  |
| Jasrotia et al., 2018 | Asian | HB | PCR-RFLP | Any Migraine | 98 | 3 | 1 | 150 | 0 | 0 | 1 |
|  |  |  |  | MA | 27 | 0 | 0 |  |  |  |  |
|  |  |  |  | MWA | 71 | 3 | 1 |  |  |  |  |
| Kaur et al., 2018 | Asian | HB | PCR-RFLP | Any Migraine | 38 | 52 | 10 | 51 | 42 | 7 | 0.97 |
|  |  |  |  | MA | 6 | 14 | 3 |  |  |  |  |
|  |  |  |  | MWA | 32 | 38 | 7 |  |  |  |  |
| Aiswarya et al., 2020 | Asian | HB | ARMS-PCR | Any Migraine | 154 | 36 | 14 | 150 | 52 | 8 | 0.61 |
|  |  |  |  | MA |  |  |  |  |  |  |  |
|  |  |  |  | MWA |  |  |  |  |  |  |  |
| Thomas et al., 2022 | Asian | HB | PCR-RFLP | Any Migraine | 107 | 44 | 35 | 89 | 44 | 19 | 0.006* |
|  |  |  |  | MA |  |  |  |  |  |  |  |
|  |  |  |  | MWA |  |  |  |  |  |  |  |

- Significant value therefore excluded from the meta-analysis

**Supplementary table-2S**1: Detail studies features exploring the MTHFR A1298Cand risk of migraine and its clinical subtype

| **Study** | **Ethnicity** | **Control** | **Tech** | **Type** |  | **Case** |  |  | **Control** |  | **HWE.P-value** |
| --- | --- | --- | --- | --- | --- | --- | --- | --- | --- | --- | --- |
|  |  |  |  |  | **HW** | **HT** | **HR** | **HW** | **HT** | **HR** |  |
| Kaur et al., 2018 | Asian | HB | PCR-RFLP | Any Migraine | 29 | 41 | 30 | 33 | 50 | 17 | 0.79 |
|  |  |  |  | MA | 6 | 11 | 6 |  |  |  |  |
|  |  |  |  | MWA | 23 | 30 | 24 |  |  |  |  |
| Thomas et al., 2022 |  |  |  | Any Migraine | 37 | 107 | 42 | 14 | 119 | 19 | 0.0001* |
|  |  |  |  | MA |  |  |  |  |  |  |  |
|  |  |  |  | MWA |  |  |  |  |  |  |  |

*Significant value therefore excluded from the meta-analysis and the variant was not eligible for meta-analysis due to only one study.

**Supplementary table-3 S**1**:** Detail studies features exploring the **ACE I/D** and risk of migraine and its clinical subtype

| **Study** | **Ethnicity** | **Control** | **Tech** | **Type** |  | **Case** |  |  | **Control** |  | **HWE.P-value** |
| --- | --- | --- | --- | --- | --- | --- | --- | --- | --- | --- | --- |
|  |  |  |  |  | **DD (Risk)** | **DI** | **II** | **DD** | **DI** | **II** |  |
| Wani et al., 2016 | Asian | HB | PCR | Any Migraine | 15 | 40 | 45 | 11 | 45 | 65 | 0.65 |
|  |  |  |  | MA | 6 | 17 | 20 |  |  |  |  |
|  |  |  |  | MWA | 9 | 23 | 25 |  |  |  |  |
| Jasrotia et al., 2018 | Asian | HB | PCR | Any Migraine | 33 | 39 | 30 | 23 | 74 | 53 | 0.73 |
|  |  |  |  | MA | 9 | 8 | 10 |  |  |  |  |
|  |  |  |  | MWA | 24 | 31 | 20 |  |  |  |  |
| Joshi et al., 2009 | Asian | HB | PCR | Any Migraine | 18 | 78 | 54 | 12 | 78 | 60 | 0.15 |
|  |  |  |  | MA | 11 | 36 | 20 |  |  |  |  |
|  |  |  |  | MWA | 7 | 42 | 34 |  |  |  |  |

**Supplementary table-4 S**1**:** Detail studies features exploring the PRDM16rs2651899and risk of migraine and its clinical subtype

| **Study** | **Ethnicity** | **Control** | **Tech** | **Type** |  | **Case** |  |  | **Control** |  | **HWE.P-value** |
| --- | --- | --- | --- | --- | --- | --- | --- | --- | --- | --- | --- |
|  |  |  |  |  | **HW** | **HT** | **HR** | **HW** | **HT** | **HR** |  |
| Ghosh et al., 2013 | Asian | HB | PCR-RFLP | Any Migraine | 108 | 149 | 82 | 41 | 106 | 53 | 0.366 |
|  |  |  |  | MA | 27 | 51 | 30 |  |  |  |  |
|  |  |  |  | MWA | 81 | 98 | 52 |  |  |  |  |
| Kaur et al., 2019 | Asian | HB | PCR-RFLP | Any Migraine | 32 | 90 | 28 | 52 | 78 | 20 | 0.366 |
|  |  |  |  | MA | 15 | 20 | 8 |  |  |  |  |
|  |  |  |  | MWA | 17 | 70 | 20 |  |  |  |  |

**Supplementary table-5 S**1**:** Detail studies features exploring the ***TRPM*8 rs10166942**and risk of migraine and its clinical subtype

| **Study** | **Ethnicity** | **Control** | **Tech** | **Type** |  | **Case** |  |  | **Control** |  | **HWE.P-value** |
| --- | --- | --- | --- | --- | --- | --- | --- | --- | --- | --- | --- |
|  |  |  |  |  | **HW** | **HT** | **HR** | **HW** | **HT** | **HR** |  |
| Ghosh et al., 2013 | Asian | HB | PCR-RFLP | Any Migraine | 57 | 175 | 101 | 44 | 91 | 65 | 0.25 |
|  |  |  |  | MA | 18 | 57 | 30 |  |  |  |  |
|  |  |  |  | MWA | 39 | 118 | 71 |  |  |  |  |
| Kaur et al., 2019 | Asian | HB | PCR-RFLP | Any Migraine | 53 | 76 | 21 | 68 | 55 | 27 | 0.01* |
|  |  |  |  | MA | 8 | 32 | 3 |  |  |  |  |
|  |  |  |  | MWA | 45 | 44 | 18 |  |  |  |  |

*Significant value therefore excluded from the meta-analysis and the variant was not eligible for meta-analysis due to only one study.

**Supplementary table-6 S**1**:** Detail studies features exploring the ESR1 PvuII and risk of migraine and its clinical subtype

| **Study** | **Ethnicity** | **Control** | **Tech** | **Type** |  | **Case** |  |  | **Control** |  | **HWE.P-value** |
| --- | --- | --- | --- | --- | --- | --- | --- | --- | --- | --- | --- |
|  |  |  |  |  | **HW** | **HT** | **HR** | **HW** | **HT** | **HR** |  |
| Joshi et al., 2010 | Asian | HB | PCR-RFLP | Any Migraine | 47 | 136 | 34 | 88 | 111 | 18 | 0.10 |
|  |  |  |  | MA | 14 | 58 | 12 |  |  |  |  |
|  |  |  |  | MWA | 33 | 78 | 22 |  |  |  |  |
| Ghosh et al., 2012 | Asian | HB | PCR-RFLP | Any Migraine | 96 | 183 | 55 | 78 | 101 | 21 | 0.16 |
|  |  |  |  | MA | 24 | 64 | 18 |  |  |  |  |
|  |  |  |  | MWA | 72 | 119 | 37 |  |  |  |  |
| Kumar et al., 2023 | Asian | HB | PCR-RFLP | Any Migraine | 48 | 42 | 12 | 53 | 56 | 6 | 0.10 |
|  |  |  |  | MA |  |  |  |  |  |  |  |
|  |  |  |  | MWA | 48 | 42 | 12 |  |  |  |  |

**Supplementary table-7 S**1**:** Detail studies features exploring the ESR XbaI and risk of migraine and its clinical subtype

| **Study** | **Ethnicity** | **Control** | **Tech** | **Type** |  | **Case** |  |  | **Control** |  | **HWE.P-value** |
| --- | --- | --- | --- | --- | --- | --- | --- | --- | --- | --- | --- |
|  |  |  |  |  | **HW** | **HT** | **HR** | **HW** | **HT** | **HR** |  |
| Gosh et al., 2012 | Asian | HB | PCR-RFLP | Any Migraine | 125 | 160 | 49 | 74 | 99 | 27 | 0.49 |
|  |  |  |  | MA |  |  |  |  |  |  |  |
|  |  |  |  | MWA |  |  |  |  |  |  |  |
| Ghosh et al., 2022 | Asian | HB | PCR-RFLP | Any Migraine | 38 | 55 | 9 | 66 | 46 | 3 | 0.25 |
|  |  |  |  | MA |  |  |  |  |  |  |  |
|  |  |  |  | MWA |  |  |  |  |  |  |  |

**Supplementary table-8 S**1**:** Detail studies features exploring the TNF 308 G>A and risk of migraine and its clinical subtype

| **Study** | **Ethnicity** | **Control** | **Tech** | **Type** |  | **Case** |  |  | **Control** |  | **HWE.P-value** |
| --- | --- | --- | --- | --- | --- | --- | --- | --- | --- | --- | --- |
|  |  |  |  |  | **HW** | **HT** | **HR** | **HW** | **HT** | **HR** |  |
| **Ghosh et al., 2010** | NI | HB | PCR-RFLP | Any Migraine | 175 | 41 | 0 | 191 | 24 | 1 | 0.79 |
|  |  |  |  | MA | 65 | 19 | 0 |  |  |  |  |
|  |  |  |  | MWA | 110 | 22 | 0 |  |  |  |  |
| **Kesavan et al., 2021** | SI | HB | PCR-RFLP | Any Migraine | 158 | 38 | 16 | 152 | 56 | 10 | 0.22 |
|  |  |  |  | MA |  |  |  |  |  |  |  |
|  |  |  |  | MWA |  |  |  |  |  |  |  |

**Supplementary table-9 S**1**:** Detail studies features exploring the LRP1**and** risk of migraine and its clinical subtype

| **Study** | **Ethnicity** | **Control** | **Tech** | **Type** |  | **Case** |  |  | **Control** |  | **HWE.P-value** |
| --- | --- | --- | --- | --- | --- | --- | --- | --- | --- | --- | --- |
|  |  |  |  |  | **HW** | **HT** | **HR** | **HW** | **HT** | **HR** |  |
| Ghosh et al., 2013 | Asian | HB | PCR-RFLP | Any Migraine | 234 | 89 | 17 | 108 | 74 | 18 | 0.61 |
|  |  |  |  | MA | 79 | 28 | 2 |  |  |  |  |
|  |  |  |  | MWA | 155 | 61 | 15 |  |  |  |  |
| Kaur et al., 2019 | Asian | HB | - | Any Migraine | 50 | 30 | 20 | 47 | 42 | 11 | 0.72 |
|  |  |  |  | MA | 14 | 5 | 4 |  |  |  |  |
|  |  |  |  | MWA | 36 | 25 | 16 |  |  |  |  |
| *Shoba et al., 2020 |  |  |  | Any Migraine | - | - | - |  |  |  |  |
|  |  |  |  | MA |  |  |  |  |  |  |  |
|  |  |  |  | MWA |  |  |  |  |  |  |  |

*Study excluded due to improper data

**Supplementary table-10 S**1**:** Detail studies features exploring the DAO-rs10156191and risk of migraine and its clinical subtype

| **Study** | **Ethnicity** | **Control** | **Tech** | **Type** |  | **Case** |  |  | **Control** |  | **HWE.P-value** |
| --- | --- | --- | --- | --- | --- | --- | --- | --- | --- | --- | --- |
|  |  |  |  |  | **HW** | **HT** | **HR** | **HW** | **HT** | **HR** |  |
| Kaur et al., 2020 | Asian | HB | PCR-RFLP | Any Migraine | 137 | 98 | 15 | 165 | 73 | 12 | 0.58 |
|  |  |  |  | MA | 35 | 30 | 5 |  |  |  |  |
|  |  |  |  | MWA | 108 | 63 | 9 |  |  |  |  |
| Thomas et al., 2022 | Asian | HB | PCR-RFLP | Any Migraine | 90 | 5 | 5 | 99 | 1 | 0 | 0.95 |
|  |  |  |  | MA |  |  |  |  |  |  |  |
|  |  |  |  | MWA |  |  |  |  |  |  |  |

**Supplementary table-11 S**1**:** Subgrouping MTHFR migraine

| **Model** | **Region** | **Number of studies** | **Test of association** | | | **Test of heterogeneity** | | | **Publication bias**  **p-val (Egger's test)** |
| --- | --- | --- | --- | --- | --- | --- | --- | --- | --- |
|  |  |  | OR | 95% CI | p-val | Model | p-val | I^2 |  |
| Allele contrast | Overall | 5 | 1.05 | [0.74-1.47] | 0.790 | Random | 0.0741 | 0.5309 | 0.3542 |
|  | NI | 4 | 1.10 | [0.67-1.80] | 0.703 | Random | 0.0404 | 0.6381 | 0.4848 |
|  | SI | 1 | 0.96 | [0.66-1.39] | 0.843 | Fixed | NA | NA | NA |
| Recessive model | Overall | 5 | 1.31 | [0.71-2.41] | 0.385 | Fixed | 0.1652 | 0.384 | 0.3253 |
|  | NI | 4 | 0.96 | [0.41-2.22] | 0.925 | Fixed | 0.1464 | 0.4417 | 0.5442 |
|  | SI | 1 | 1.86 | [0.76-4.53] | 0.172 | Fixed | NA | NA | NA |
| Dominant model | Overall | 5 | 1.08 | [0.72-1.61] | 0.710 | Random | 0.073 | 0.5329 | 0.2371 |
|  | NI | 4 | 1.22 | [0.72-2.06] | 0.460 | Random | 0.0789 | 0.5582 | 0.3937 |
|  | SI | 1 | 0.81 | [0.52-1.25] | 0.350 | Fixed | NA | NA | NA |
| Over-dominant | Overall | 5 | 1.06 | [0.70-1.60] | 0.787 | Random | 0.0761 | 0.5272 | 0.2918 |
|  | NI | 4 | 1.24 | [0.90-1.69] | 0.187 | Fixed | 0.3045 | 0.1731 | 0.3151 |
|  | SI | 1 | 0.65 | [0.40-1.04] | 0.078 | Fixed | NA | NA | NA |

**NI:** North India, **SI:** South India

**Supplementary table-12 S**1**:**ESR1 XbaI: Overall association of XbaI variant of *ESR1* with the risk of migraine and its subtypes

| **Type** | **Model** | **Number of studies** | **Test of association** | | | **Test of heterogeneity** | | | **Publication bias**  **p-val (Egger's test)** |
| --- | --- | --- | --- | --- | --- | --- | --- | --- | --- |
|  |  |  | **OR** | **95% CI** | **p-val** | **Model** | **p-val** | **I^2^** |  |
| **Migraine** | Allele | 2 | 1.36 | 0.73- 2.5 | 0.32 | R | 0.0121 | 0.8411 | NaN |
|  | Recessive | 2 | 1.27 | 0.79- 2.05 | 0.30 | F | 0.1031 | 0.6237 | NaN |
|  | Dominant | 2 | 1.45 | 0.6- 3.2 | 0.36 | R | 0.0122 | 0.8406 | NaN |
|  | Over-dominant | 2 | 1.23 | 0.6- 2.2 | 0.49 | R | 0.0562 | 0.7257 | NaN |
| **MWA** |  |  |  |  |  |  |  |  |  |
|  | Allele | 2 | 1.32 | 0.6- 2.5 | 0.40 | R | 0.0076 | 0.8599 | NaN |
|  | Recessive | 2 | 1.66 | 0.5- 5.3 | 0.39 | R | 0.0927 | 0.6463 | NaN |
|  | Dominant | 2 | 1.40 | 0.5- 3.4 | 0.46 | R | 0.007 | 0.8626 | NaN |
|  | Over-dominant | 2 | 1.21 | 0.6- 2.3 | 0.57 | R | 0.0424 | 0.7572 | NaN |
| **MA** | Allele | 2 | 1.30 | 0.96-1.75 | 0.09 | F | 0.12 | 0.57 | NaN |
|  | Recessive | 2 | 1.35 | 0.72-2.51 | 0.34 | F | 0.37 | 0 | NaN |
|  | Dominant | 2 | 1.45 | 0.94-2.23 | 0.09 | F | 0.11 | 0.59 | NaN |
|  | Over-dominant | 2 | 1.25 | 0.83-1.89 | 0.28 | F | 0.14 | 0.52 | NaN |

**Supplementary table-13 S**1**:** Overall association of PRDM-16 and risk of migraine

| Clinical type | Model | Study | Number of studies | Test of association | | | Test of heterogeneity | | | Publication bias  p-val (Egger's test) |
| --- | --- | --- | --- | --- | --- | --- | --- | --- | --- | --- |
|  |  |  |  | OR | 95% CI | p-val | Model | p-val | I^2 |  |
| Migraine | Allele contrast | Overall | 2 | 1.05 | [0.55-1.98] | 0.8919374 | Random | 0.0017 | 0.8987 | NaN |
|  | Recessive model | Overall | 2 | 1.03 | [0.73-1.44] | 0.8620778 | Fixed | 0.1678 | 0.4743 | NaN |
|  | Dominant model | Overall | 2 | 1.03 | [0.29-3.55] | 0.9643928 | Random | 0.0002 | 0.9293 | NaN |
|  | Over-dominant | Overall | 2 | 0.97 | [0.49-1.89] | 0.9184995 | Random | 0.0192 | 0.8175 | NaN |
| MA | Allele contrast | Overall | 2 | 0.99 | [0.75-1.30] | 0.9373116 | Fixed | 0.573 | 0 | NaN |
|  | Recessive model | Overall | 2 | 1.16 | [0.73-1.82] | 0.5207561 | Fixed | 0.5335 | 0 | NaN |
|  | Dominant model | Overall | 2 | 0.85 | [0.54-1.31] | 0.4642733 | Fixed | 0.5913 | 0 | NaN |
|  | Over-dominant | Overall | 2 | 0.80 | [0.54-1.17] | 0.2476136 | Fixed | 0.9781 | 0 | NaN |
| MWA | Allele contrast | Overall | 2 | 1.05 | [0.45-2.44] | 0.9068959 | Random | 0.0001 | 0.9307 | NaN |
|  | Recessive model | Overall | 2 | 0.97 | [0.66-1.40] | 0.8641467 | Fixed | 0.1338 | 0.5552 | NaN |
|  | Dominant model | Overall | 2 | 1.14 | [0.20-6.48] | 0.8807991 | Random | 0 | 0.9525 | NaN |
|  | Over-dominant | Overall | 2 | 1.05 | [0.40-2.75] | 0.9179185 | Random | 0.0025 | 0.8905 | NaN |

**Supplementary table-14 S**1: Overall association of ACE I/D and risk of migraine

| **Model** | **Ethnicity** | **Number of studies** |  | **Test of association** |  |  | **Test of heterogeneity** |  | **Publication bias** |
| --- | --- | --- | --- | --- | --- | --- | --- | --- | --- |
|  |  |  | **OR** | **95% CI** | **p-val** | **Model** | **p-val** | **I^2** | **p-val (Egger's test)** |
| **Allele** | Overall | 3 | 1.3733 | [1.1141; 1.6929] | 0.002956 | Fixed | 0.5044 | 0 | 0.6801 |
| **Recessive** | Overall | 3 | 2.0547 | [1.3594; 3.1056] | 0.000634 | Fixed | 0.5326 | 0 | 0.2619 |
| **Dominant** | Overall | 3 | 1.2903 | [0.9612; 1.7322] | 0.089779 | Fixed | 0.8812 | 0 | 0.3354 |
| **Overdominant** | Overall | 3 | 0.8958 | [0.6717; 1.1946] | 0.453612 | Fixed | 0.2686 | 0.2393 | 0.9617 |

**Supplementary table-15 S**1: Overall association of TNF A -308 G>A and risk of migraine

| **Model** | **Ethnicity** | **Number of studies** |  | **Test of association** |  |  | **Test of heterogeneity** |  | **Publication bias** |
| --- | --- | --- | --- | --- | --- | --- | --- | --- | --- |
|  |  |  | **OR** | **95% CI** | **p-val** | **Model** | **p-val** | **I^2** | **p-val (Egger's test)** |
| **Allele** | Overall | 2 | 1.2004 | [0.6968; 2.0681] | 0.510412 | Random | 0.0787 | 0.6765 | NaN |
|  | NI | 1 | 1.6374 | [0.9827; 2.7285] | 0.058383 | Fixed | NA | NA | NA |
|  | SI | 1 | 0.9367 | [0.6559; 1.3376] | 0.718897 | Fixed | NA | NA | NA |
| **Recessive** | Overall | 2 | 1.5382 | [0.6989; 3.3852] | 0.284653 | Fixed | 0.3333 | 0 | NaN |
|  | NI | 1 | 0.3318 | [0.0134; 8.1900] | 0.500041 | Fixed | NA | NA | NA |
|  | SI | 1 | 1.6980 | [0.7525; 3.8315] | 0.202294 | Fixed | NA | NA | NA |
| **Dominant** | Overall | 2 | 1.1664 | [0.5218; 2.6072] | 0.707677 | Random | 0.0186 | 0.8194 | NaN |
|  | NI | 1 | 1.7899 | [1.0452; 3.0653] | 0.033919 | Fixed | NA | NA | NA |
|  | SI | 1 | 0.7871 | [0.5156; 1.2016] | 0.267348 | Fixed | NA | NA | NA |
| **Overdominant** | Overall | 2 | 1.0778 | [0.3713; 3.1281] | 0.890421 | Random | 0.0029 | 0.8875 | NaN |
|  | NI | 1 | 1.8743 | [1.0881; 3.2285] | 0.023557 | Fixed | NA | NA | NA |
|  | SI | 1 | 0.6318 | [0.3972; 1.0050] | 0.052505 | Fixed | NA | NA | NA |
